# The Elongator Complex Interacts with PCNA and Modulates Transcriptional Silencing and Sensitivity to DNA Damage Agents

**DOI:** 10.1371/journal.pgen.1000684

**Published:** 2009-10-16

**Authors:** Qing Li, A. M. Fazly, Hui Zhou, Shengbing Huang, Zhiguo Zhang, Bruce Stillman

**Affiliations:** 1 Department of Biochemistry and Molecular Biology, Mayo Clinic College of Medicine, Rochester, Minnesota, United States of America; 2 Cold Spring Harbor Laboratory, Cold Spring Harbor, New York, United States of America; The Hospital for Sick Children and University of Toronto, Canada

## Abstract

Histone chaperones CAF-1 and Asf1 function to deposit newly synthesized histones onto replicating DNA to promote nucleosome formation in a proliferating cell nuclear antigen (PCNA) dependent process. The DNA replication- or DNA repair-coupled nucleosome assembly pathways are important for maintenance of transcriptional gene silencing and genome stability. However, how these pathways are regulated is not well understood. Here we report an interaction between the Elongator histone acetyltransferase and the proliferating cell nuclear antigen. Cells lacking Elp3 (K-acetyltransferase Kat9), the catalytic subunit of the six-subunit Elongator complex, partially lose silencing of reporter genes at the chromosome VIIL telomere and at the *HMR* locus, and are sensitive to the DNA replication inhibitor hydroxyurea (HU) and the damaging agent methyl methanesulfonate (MMS). Like deletion of the *ELP3*, mutation of each of the four other subunits of the Elongator complex as well as mutations in Elp3 that compromise the formation of the Elongator complex also result in loss of silencing and increased HU sensitivity. Moreover, Elp3 is required for S-phase progression in the presence of HU. Epistasis analysis indicates that the *elp3*Δ mutant, which itself is sensitive to MMS, exacerbates the MMS sensitivity of cells lacking histone chaperones Asf1, CAF-1 and the H3 lysine 56 acetyltransferase Rtt109. The *elp3*Δ mutant has allele specific genetic interactions with mutations in *POL30* that encodes PCNA and PCNA binds to the Elongator complex both *in vivo* and *in vitro*. Together, these results uncover a novel role for the intact Elongator complex in transcriptional silencing and maintenance of genome stability, and it does so in a pathway linked to the DNA replication and DNA repair protein PCNA.

## Author Summary

During S phase of the cell cycle, not only must DNA sequences be faithfully duplicated, chromatin structures must also be inherited into daughter cells to maintain gene expression states and cell identity. While significant progress has been made in understanding the regulation of DNA replication, how chromatin structures are maintained from one cell division cycle to the next (so-called epigenetic inheritance) is only partially understood. It is believed that the DNA replication-coupled nucleosome assembly process plays an important role in such inheritance as well as maintenance of genome stability. In this process, histone chaperones such as chromatin assembly factor 1 (CAF-1) deposit newly synthesized histones H3–H4, which are acetylated at specific lysine residues, onto replicating DNA in a PCNA dependent reaction. PCNA is a clamp for DNA polymerases and other proteins that are involved in DNA replication and DNA repair. Genetic interactions between lysine acetyltransferase Elp3 and factors involved in DNA replication-coupled nucleosome assembly are described. Elp3 is required for transcriptional silencing and for maintenance of genome stability and binds directly to PCNA. A role for the Elongator complex in response to DNA damage and in maintenance of gene silencing is discussed.

## Introduction

Chromatin, an organized complex of proteins and DNA in eukaryotic cells, is regulated by diverse means to govern cellular processes including gene transcription, DNA replication and DNA repair. For instance, histones that form the core components of nucleosomes, the basic building block of chromatin, are modified post-translationally by acetylation, methylation, phosphorylation and ubiquitination. Distinct modifications of histones can either alter chromatin structure and/or recruit proteins to chromatin to impact a specific cellular process [1]–[3]. In addition, histone chaperones facilitate DNA replication and gene transcription processes by binding to histones for nucleosome assembly and/or disassembly [4]. Furthermore, chromatin can be remodeled by ATP dependent chromatin remodeling machines [5]. Emerging evidence suggests that histone modifications, nucleosome assembly and chromatin remodeling coordinate to regulate chromatin structures during gene transcription, DNA replication and DNA repair and other cellular processes.

Elp3 is the catalytic subunit of the Elongator histone acetyltransferase (HAT) that was originally purified as a component preferentially associating with hyperphosphorylated forms of RNA polymerase II [6]. The *ELP* genes were also identified as the target of zymocin, a three-subunit toxin complex secreted by *Kluyveromyces lactis* that inhibits cell cycle progression of *S. cerevisiae* [7]. Elp3 forms a stable complex with five other polypetides called Elp1, Elp2, Elp4, Elp5 and Elp6 [8], and contains two domains. The C-terminal domain of Elp3 is similar to the catalytic domain of the Gcn5 HAT and the purified Elp3 complexes have acetyltransferase activities for free and nucleosomal histones H3 and H4, with lysine 14 of H3 and lysine 8 of H4 as the prime targets [9]. Elp3 has been named Kat9 K-acetyltransferase to conform with recent acetyltransferase nomenclature [10]. Elp3 also contains a region that bears sequence similarity to the catalytic domain of Radical SAM enzymes. Indeed, the corresponding domain of *M. jannaschii* Elp3 binds S-adenosylmethionine (SAM) *in vitro* and contains an iron-sulphur cluster, two characteristics of Radical SAM enzymes [11]. The function of this motif, however, is not known.

Elp3 is localized predominantly in the cytosol suggesting that the Elongator complex has functions other than transcription elongation. Budding yeast cells lacking Elp3 exhibit severe growth defects in the absence of Gcn5 [12], the catalytic subunit of the SAGA and ADA histone acetyltransferases [5],[13]. Silent information regulator proteins (Sir) that are structural components of heterochromatin in budding yeast are found within euchromatin in cells lacking both Elp3 and Gcn5, suggesting that hypoacetylation of histones affects the integrity of telomeric chromatin in the yeast *S. cerevisiae* [12]. However, it is not clear whether Elp3 is also important for silencing at other silent chromatin loci such as the two silent mating type loci, *HML* and *HMR*. In addition, the Elongator complex has been shown to be required for wobble uridine tRNA modification [14]. Recently, the mammalian Elongator complex was shown to acetylate α-tubulin and stabilize microtubules [15]. Finally, the Elongator has been implicated in regulation of polarized exocytosis [16]. These results indicate that the conserved Elongator complex functions in multiple cellular processes.

Histone chaperones are a group of proteins that bind histones and promote nucleosome assembly during DNA replication, DNA repair and gene transcription [4]. The classical histone chaperone involved in DNA replication-coupled nucleosome assembly is Chromatin Assembly Factor 1 (CAF-1) [17],[18]. CAF-1 deposits newly synthesized histone H3–H4 onto replicating DNA to promote nucleosome formation. The ability of CAF-1 to deposit histones preferentially onto replicating DNA is mediated through the physical interactions between CAF-1 and the Proliferating Cell Nuclear Antigen (PCNA) [19],[20]. PCNA is a protein clamp that is loaded onto DNA at the replication fork or at sites of DNA repair by an ATPase called RFC (Replication Factor C) and interacts with many proteins involved in DNA replication and DNA repair, including the replicative DNA polymerases [21]. In addition to CAF-1 and PCNA, Asf1, first identified as a factor disrupting heterochromatin silencing when over-expressed in yeast [22], is also involved in DNA replication-coupled nucleosome assembly [23]–[27]. Recent studies indicate that the ability of Asf1 to promote replication-coupled nucleosome assembly is mediated through its ability to regulate acetylation of lysine 56 of H3 [28],[29]. Lysine 56 on H3 is localized at DNA entry and exit points of a nucleosome [30]. Acetylation of this lysine residue is catalyzed by histone acetyltransferase Rtt109 (Kat11) and is dependent on the presence of Asf1 in yeast cells [31]–[37]. Moreover, this modification increases the binding of H3 with CAF-1 and subsequent assembly of new H3–H4 onto replicating DNA [29]. Thus, Asf1 and CAF-1 cooperate to assemble specifically modified histones during DNA synthesis.

In yeast cells, factors such as CAF-1, Asf1 and PCNA that are known to be involved in DNA replication-coupled nucleosome assembly are required for both transcriptional gene silencing as well as maintenance of genome integrity [22], [23], [38]–[41]. For instance, mutations in CAF-1, Asf1 or PCNA result in partial loss of transcriptional silencing and increased sensitivity towards DNA damaging agents [23],[42],[43]. Genetic and biochemical evidence indicate that the roles of Asf1 in transcriptional silencing and maintenance of genome stability are separable. For instance, the function of Asf1 in transcriptional silencing is linked to its interaction with Hir1 [44], a histone chaperone that is involved in DNA replication-independent nucleosome assembly. On the other hand, recent studies have shown that the ability of Asf1 to maintain genome stability is mediated mainly through Asf1's role in regulating acetylation of lysine 56 of H3 [31],[33],[36],[37],[45]. Moreover different PCNA mutant alleles exhibit distinct effects on transcriptional silencing and sensitivity to DNA damaging agents [43]. Thus, it is possible that the functions of CAF-1 and PCNA in transcriptional silencing and maintenance of genome stability are also mediated through their interactions with different partners or pathways.

In both yeast and human cells CAF-1 binds histones H3/H4 in which histone H4 is acetylated on lysine residues 5, 8 and 12 [46],[47]. Acetylation of lysine residues 5 (H4K5) and 12 (H4K12) of H4 is catalyzed by the Hat1 histone acetyltransferase; the HAT responsible for acetylation of lysine 8 (H4K8) is not known although Elongator can acetylate H4K8 and histone H3 lysine 14 *in vitro* [9]. Interestingly, Asf1 has been shown to co-purify with Hat1 [48]. In addition to Hat1, CAF-1 and Asf1 interact with Sas2 physically and genetically [49],[50]. Sas2, the catalytic subunit of SAS histone acetyltransferase, acetylates lysine 16 of H4 and is involved in silencing at telomere and the *HM* loci [51],[52]. However, the functions of these interactions are not clear. Thus, factors involved in DNA replication-coupled nucleosome assembly pathway also interact with multiple HATs. In this report, we describe genetic and biochemical interactions between Elp3 and factors involved in nucleosome assembly pathways and DNA replication and repair. Cells lacking Elp3 are defective in transcriptional silencing and are sensitive to DNA damaging agents including hydroxyurea and MMS, two phenotypes shared by cells containing mutations in genes known to be involved in DNA replication-coupled nucleosome assembly. Moreover, we have shown that mutations at the SAM binding module of Elp3 abolish its interactions with Elp2 and subsequent formation of an intact Elongator complex. Biochemical analysis suggests that Elp3 is one of the HATs responsible for acetylating H4K8 *in vivo* and genetic analysis shows interactions between Elp3 and proteins involved in replication-coupled nucleosome assembly. Lastly, the Elongator complex physically interacts with PCNA. These results reveal a novel role of the Elongator in maintenance of genome stability.

## Results

### Cells lacking Elp3 are partially defective in transcriptional silencing at both telomeres and the *HMR* locus and are sensitive to DNA damaging agents

**Figure 1:**
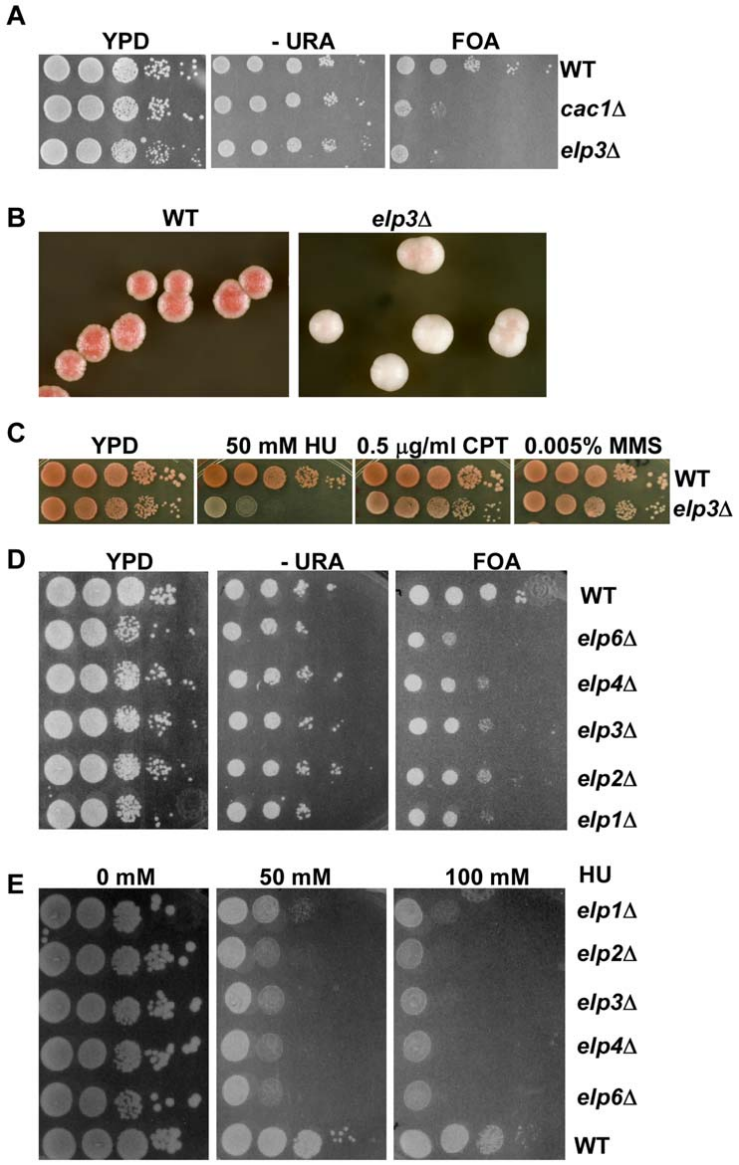
The *elp3*Δ mutant is partially defective in silencing at telomeres and at the *HMR* locus and sensitive to DNA damage agents. (A) The *elp3*Δ mutant cells partially lose telomeric silencing. Ten-fold serial dilutions of yeast cells with indicated genotypes were spotted onto non-selective medium to assay cell growth, medium lacking uracil (-URA) or containing FOA to assay telomeric silencing. (B) The *elp3*Δ mutant is partially defective in silencing at the *HMR* locus. Wild type or *elp3*Δ mutant cells in which the *ADE2* gene was integrated at the *HMR* locus were spread onto YPD medium. After growth at 30°C for 3 days, the plates were incubated at 4°C for one day before pictures were taken using a digital camera. (C) The *elp3*Δ mutant is sensitive to DNA damage agent HU. Ten-fold serial dilutions of wild type and *elp3*Δ mutant cells were plated on non-selective YPD medium or YPD medium containing indicated concentrations of DNA damaging agents MMS, HU and CPT. A more complete presentation of the data is shown in Figure S1. (D,E) Cells lacking other subunits of the Elp3 containing Elongator complex display similar defects in telomeric silencing (D) and sensitivity towards HU to the *elp3*Δ mutant cells (E). Wild type or each mutant strain with indicated genotypes were tested for telomeric silencing and HU sensitivity as described in A and C.

Cells lacking Elp3 and Gcn5 are synthetic lethal. Deletion of Sir3, the protein component of yeast silent chromatin suppresses the synthetic lethality of the *gcn5*Δ*elp3*Δ cells that may arise from the spreading of Sir proteins from telomeric silent chromatin to telomeric euchromatin in *gcn5*Δ*elp3*Δ mutant cells [12]. These results suggest that Elp3 may have a role in the maintenance of telomeric gene silencing in the yeast *S. cerevisiae* or yield a phenotype that is sensitive to the state of telomeric silencing. To test this idea, the *URA3* gene integrated near the left telomere of chromosome VII was used as a reporter gene [53],[54]. Silencing of the *URA3* displayed variegated gene expression because a fraction of wild type cells expressed the *URA3* gene, whereas the *URA3* gene is repressed in the rest of the cell population. Therefore, a mixed population of wild type cells grew efficiently in medium lacking uracil, an indication of expression of the *URA3* gene, and also in medium containing the drug FOA that killed cells expressing the *URA3* gene (Figure 1A). Compared to wild type cells, cells lacking Cac1, the large subunit of CAF-1 that is known to be involved in telomeric silencing [38], grew very poorly in the FOA medium, an indication of reduction in telomeric silencing in the *cac1*Δ mutant cells (Figure 1A). Cells lacking the Elp3 also grew poorly on FOA containing medium, suggesting a significant reduction in telomeric gene silencing compared to wild type cells, but to a lesser extent than cells lacking Cac1 (Figure 1A and data not shown). This result suggests that Elp3 affects efficient telomeric gene silencing of the *URA3* reporter gene.

Next, we tested whether Elp3 depletion alters silencing at the silent mating type locus *HMR* using the yeast colony color assay in which the reporter gene *ADE2* was integrated at the *HMR* locus. This assay is very sensitive to detect defects in *HMR* silencing, more so than the endogenous *HMR* genes [55]. In wild type cells, the *ADE2* gene was silenced and thus the colony color was red (Figure 1B). However, if silencing of the *ADE2* gene was completely lost due to mutations at genes essential for silencing at the *HMR* locus, the colony color would be white [55]. Interestingly, the *elp3*Δ mutant cells exhibited red/white sectoring colonies with predominant white appearance (Figure 1B), suggesting that stable inheritance of silencing of the *HMR*-linked reporter gene was compromised in the *elp3*Δ mutant cells. However, when assayed with the *GFP* transgene transcribed from a different promoter integrated at the *HMR* locus, we could not detect much effect of the *elp3*Δ mutation on silencing of *GFP* at this locus (Table S1). This phenotype is similar to that of the *cac1*Δ mutant cells. The *cac1*Δ mutant cells also display *HMR* silencing defect when assayed with the *ADE2* gene at the *HMR* locus [43], however the effect of the *cac1*Δ mutation on silencing at the *HMR* locus was barely detectable using the *GFP* reporter gene [56]. Together, these results indicate that Elp3 controls reporter gene expression at both telomeres and at the *HMR* locus, but the results depend on the reporter nature of the gene.

We also tested whether cells lacking Elp3 were sensitive to DNA damaging agents methyl methanesulfonate (MMS, a DNA alkylating agent), hydroxyurea (HU, an inhibitor of ribonucleotide reductase), and camptothecin (CPT, an inhibitor of topoisomerase I). The *elp3*Δ mutant cells were slightly sensitive to 0.01% MMS and were very sensitive to the drug HU that depletes dNTP pools and slows down DNA replication (Figure 1C and Figure S1A, S1B, S1C). On the other hand, CPT had no effect on the growth of *elp3*Δ cells. These results suggest that Elp3 is critical in protecting cells from the insult of HU that slows down DNA replication forks and from DNA damage induced during DNA replication.

The Elp3 protein is the catalytic subunit of the Elongator complex that consists of Elp1–Elp6. While five subunits (Elp1–Elp4 and Elp6) were not essential in all conditions tested, deleting the *ELP5* gene was essential under certain conditions [8]. We therefore tested whether cells lacking each of the five non-essential subunits were defective in telomeric silencing and sensitive to HU. Cells lacking each of the five subunits exhibited similar defects in telomeric silencing (Figure 1D). Moreover, each mutant strain displayed similar degrees of sensitivity towards HU (Figure 1E) and bleomycin (Figure S1D), an agent that causes double strand breaks in cells. Thus, five subunits of the Elongator complex are required for efficient telomeric silencing and resistance to killing by DNA damaging agents, suggesting that an intact Elongator complex functions in these processes.

### Cells expressing *elp3* mutants with mutations in the HAT domain and the Radical SAM domain of Elp3 are defective in silencing and sensitive to DNA damaging agents

**Figure 2:**
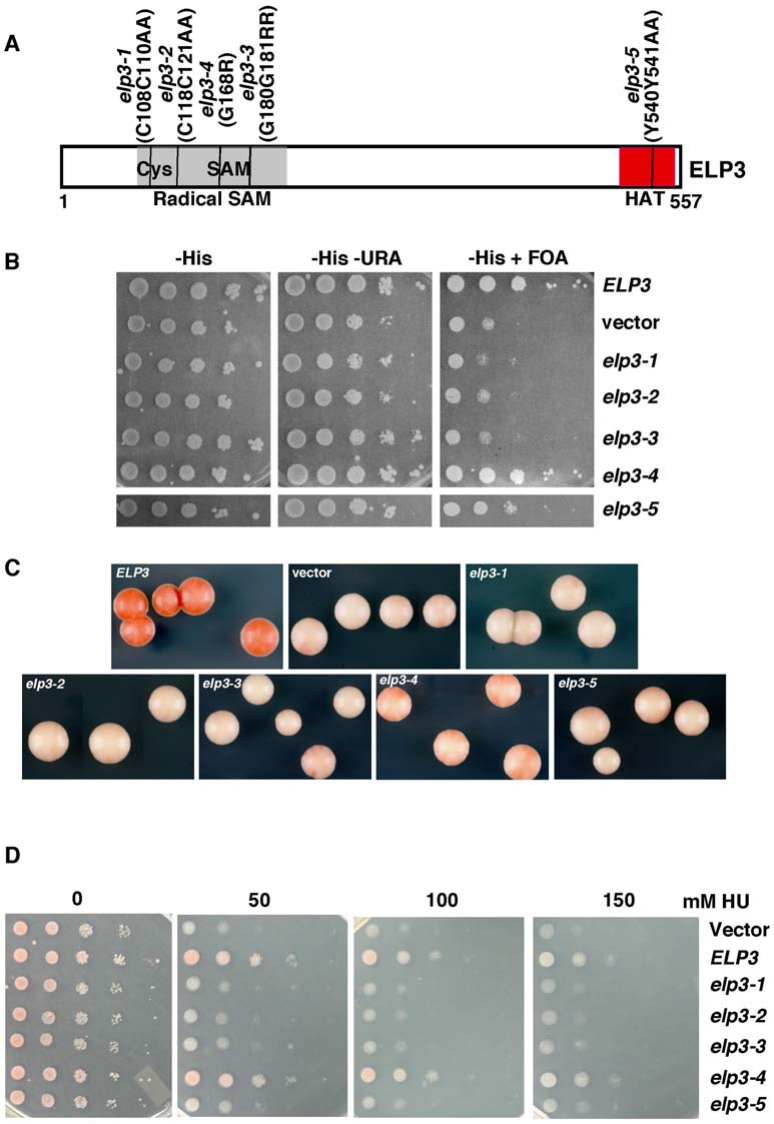
Cells expressing *elp3* site-specific mutants with mutations at the HAT domain and the conserved Radical SAM domain of Elp3 reduce transcriptional silencing and are sensitive to HU. (A) A schematic representation of mutations on Elp3. Highlighted are the domains that bind S-adenosylmethionine (SAM) and the signature of histone acetyltransferase (HAT). Several amino acids located in the Radical SAM domains were mutated and these included *elp3-1* with cysteine residues 108 and 110 replaced with alanine; *elp3-2* with cysteine residues 118 and 121 replaced with alanine; *elp3-4* with glycine 168 replaced with an arginine; *elp3-3* with glycine 180 and 181 replaced with arginine. Two tyrosine residues Tyr 540 and 541 located at HAT domain were also replaced with alanine (*elp3-5*). (B) Four of the five site-specific *elp3*Δ mutants reduce telomeric silencing. Plasmids expressing wild type or each of the *elp3* mutants were transformed into the *elp3*Δ mutant cells and telomeric silencing was assayed as described in Figure 1. (C) All five *elp3* mutants reduce silencing of the *ADE2* gene at telomeres. The *ADE2* gene was integrated at the *HMR* locus and used to assay silencing of each of the five site-specific *elp3*Δ mutants. (D) The site-specific *elp3* mutant cells are also sensitive to HU. Ten fold serial dilutions of yeast cells expressing wild type or each of the five site-specific *elp3* mutants were spotted onto different concentrations of HU media as indicated.

Elp3 contains a region that is similar to the catalytic domain of other HATs, and mutations altering two tyrosine residues within this domain result in reduced HAT activity [9]. In addition to the HAT domain, a region from residues 77 to 363 is similar to the catalytic domain of Radical SAM enzymes. Moreover, the corresponding region of Elp3 from *M. jannaschii* has been shown to bind SAM and contains an iron-sulphur cluster. We therefore tested whether the HAT domain and the Radical SAM domain were required for transcriptional silencing of reporter genes at *HMR* and telomeres and for HU resistance. Two tyrosine residues (540 and 541 located within the HAT domain) were replaced with alanine (*elp3-5*) (Figure 2A), and several residues were changed that have been implicated to be critical for the formation of iron-sulfur cluster or SAM binding in other enzymes within the Radical SAM domain of Elp3: *elp3-1* (containing mutations at two cysteine residues 108 and 110); *elp3-2* (containing mutations at two cysteine residues 118 and 121); *elp3-3* (containing mutations at two glycine residues 180 and 181) and *elp3-4* (containing a mutation at glycine 168) (Figure 2A). These mutant alleles and wild type *ELP3* were expressed in the *elp3*Δ cells and their effects on telomeric silencing were determined (Figure 2B). Wild type Elp3 expressed from the plasmid complemented the silencing defect of the *elp3*Δ mutant cells (Figure 2B and data not shown). Three *elp3* mutants, the *elp3-1*, *elp3-2* and *elp3-3* reduced telomeric gene silencing to a degree similar to the *elp3*Δ mutant. As detailed below, these mutant alleles affect the structural integrity of the Elongator complex. By contrast, the *elp3-5* mutant reduced telomeric gene silencing less than the *elp3*Δ mutant, whereas the *elp3-4* mutant had no detectable effect on telomeric gene silencing. Thus, mutations within the HAT domain had a lesser effect on telomeric gene silencing than the *elp3*Δ mutant, whereas three of four mutations within the Radical SAM domain reduced telomeric silencing to a similar degree as the *elp3*Δ mutant.

The effects of these mutant alleles were also tested on silencing at the *HMR* locus (Figure 2C). All of these mutations reduced silencing of the *ADE2* reporter gene to a significant degree compared to wild type cells. Three mutant alleles, *elp3-1*, *elp3-2*, *elp3-3* reduced silencing of the *ADE2* gene at the *HMR* locus to a similar degree as the *elp3*Δ mutant, whereas the *elp3-5* mutant allele reduced silencing at the *HMR* locus to a lesser degree compared to other site-specific *elp3* mutants. The *elp3-4* mutant had the weakest effect, but nonetheless reduced silencing compared to wild type by producing sectoring cells (Figure 2C and data not shown). Together, these results indicate that HAT domain and the Radical SAM domain of Elp3 are required for silencing at the *HMR* locus.

Lastly, the site-specific *elp3* mutant cells were tested for their response to the HU treatment (Figure 2D). The three *elp3* mutants (*elp3-1*, *elp3-2 and elp3-3*) were as sensitive to HU as the *elp3*Δ mutant, whereas the *elp3-5* mutant was less sensitive to HU than the *elp3*Δ mutant and the *elp3-4* mutant had no effect. Thus, the HU sensitivity of these five *elp3* site-specific mutants was similar to the telomeric gene silencing defect displayed by these mutants.

### Effect of *ELP3* mutations within the Radical SAM domain on Elongator complex stability

**Figure 3:**
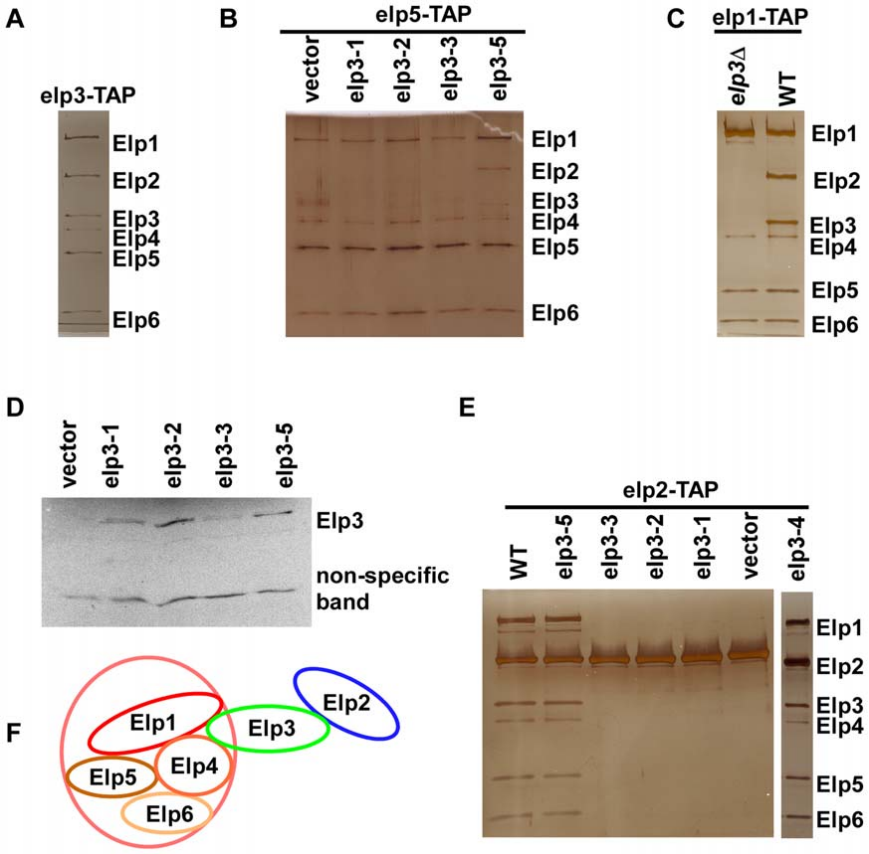
Mutations at the SAM domain, but not at the HAT domain, of *elp3* perturb the integrity of the Elongator complex. (A) Elp3 forms a complex with five other subunits of Elongator. The Elongator complex was purified from cells expressing Elp3-TAP and the purified proteins were visualized by silver staining. (B) Three *elp3* site-specific mutants affect formation of the Elongator complex. Plasmids for wild type Elp3, *elp3-1*, *elp3-2*, *elp3-3* and *elp3-5* were introduced into the strain in which the *ELP3* was deleted and *ELP5* was tagged with the TAP epitope (*elp3*Δ *elp5-TAP*). Proteins associated with Elp5 were purified, resolved on SDS PAGE and revealed with silver staining. As a negative control, Elp5 was also purified from a strain transformed with empty vector (vector). (C) Elp1 molecules associate with Elp4–6 subcomplex in the absence of Elp3. Elp1 molecules were purified from wild type or *elp3*Δ mutant cells using the TAP tag and purified proteins were analyzed on SDS-PAGE and visualized by silver staining. (D) Analysis of expression of *elp3* mutants by Western blot. Whole cell extracts from cells expressing wild type Elp3 or each of the site-specific *elp3* mutants were analyzed by Western blot using antibodies against the Flag epitope, which was fused to the C-terminus of Elp3 or each site-specific mutant of Elp3. (E) Mutations in the Radical SAM domain of Elp3 affect the binding of Elp3 to Elp2. Elp2 proteins were purified from yeast cells expressing wild type Elp3 and each of the four site-specific *elp3* mutants (*elp3-1*, *elp3-2*, *elp3-3* and *elp3-5*) using Elp2-TAP and co-purified proteins were detected by silver staining. (F) A model of the subunit organization of the Elongator complex highlighting the interaction between Elp2 and Elp3 is important for Elp2 to incorporate into the Elongator complex and the Elp1, Elp4, Elp5, and Elp6 proteins form a core complex.

Elp3 forms a complex with five other polypetides, Elp1, Elp2, Elp4, Elp5 and Elp6 [8]. The Elongator complex could be purified from yeast cells by affinity purification using Elp3 fused at its C-terminus with the TAP tag that contains both a protein-A binding module and a calmodulin binding peptide (CBP) separated by a TEV cleavage site (Figure 3A and 3F). Mutational analysis on the phenotypes of each of the five subunits of the Elongator complex suggests that the integral Elongator complex is required for silencing and resistance to DNA damage agents. Thus, it is possible that the silencing defect and sensitivity towards DNA damage displayed by the five *elp3* site-specific mutants were due to compromised formation of the Elongator complex. To test this possibility, each mutant allele was expressed in a yeast strain in which the *ELP3* gene was deleted and the Elp5 subunit was tagged with the TAP epitope (*elp3*Δ, *Elp5-TAP*). The six subunits of the ELP complex were purified from the *elp3-5* mutant cells using the Elp5-TAP (Figure 3B), indicating that this *elp3* mutant did not affect formation of the Elongator complex. Thus, the silencing defects and HU sensitivity displayed by the *elp3-5* mutant, which contain mutations within the HAT domain of Elp3, were most likely due to inactivation of HAT activity of this enzyme. In contrast, only four subunits, Elp1, Elp4, Elp5 and Elp6 were purified from cells expressing the *elp3-1*, *elp3-2*, or *elp3-3* mutant. These results indicate that the silencing defects and HU sensitivity of these three *elp3* site-specific mutant cells may be due to loss of an intact six subunit Elongator complex.

In yeast cells, the Elongator complex can be subdivided into two subcomplexes: Elp1, Elp2 and Elp3, and Elp4, Elp5 and Elp6 [8]. It has also been reported that Elp1 binds to the Elp4–6 subcomplex through its interaction with Elp3 [57]. The latter result directly contradicts our observation that Elp1 forms a complex with Elp4–Elp6 in the absence of Elp3 (Figure 3B). To test whether Elp1 binds to Elp4–Elp6 subcomplexes, we purified Elp1 using Elp1-TAP from wild type and *elp3*Δ mutant cells. As shown in Figure 3C, Elp1 co-purified with Elp4–Elp6 in the absence of Elp3. Thus, Elp1 forms a complex with the Elp4–Elp6 subcomplex *in vivo*.

Elp2 did not co-purify with Elp1, Elp4, Elp5, and Elp6 in cells lacking Elp3 (Figure 3C), suggesting that Elp2 interacted with Elp3 and that this interaction was important for Elp2 to be incorporated into an intact Elongator complex. Moreover, Elp2 did not co-purify with Elp5 from cells expressing the three *elp3* site-specific mutants (*elp3-1*, *elp3-2* and *elp3-3*) that contain mutations at the Radical SAM domain (Figure 3B). Because these three mutant proteins were expressed at similar levels (Figure 3D), this result suggested that mutations at the radical SAM domain of Elp3 affected the binding of Elp2 to the Elp complex. To test this idea further, Elp2 was purified from *elp3-1*, *elp3-2*, *elp3-3* and *elp3-5* mutant cells. While the intact Elp1–Elp6 complex was purified from *elp3-4* and *elp3-5* cells, only Elp2 was purified from *elp3-1*, *elp3-2* and *elp3-3* mutant cells (Figure 3E). These results suggest that the radical SAM domain of Elp3 may mediate the interaction between Elp3 and Elp2 and subsequent incorporation of Elp2 into the Elongator complex (Figure 3F).

### Elp3 is required for S-phase progression in the presence of HU

**Figure 4:**
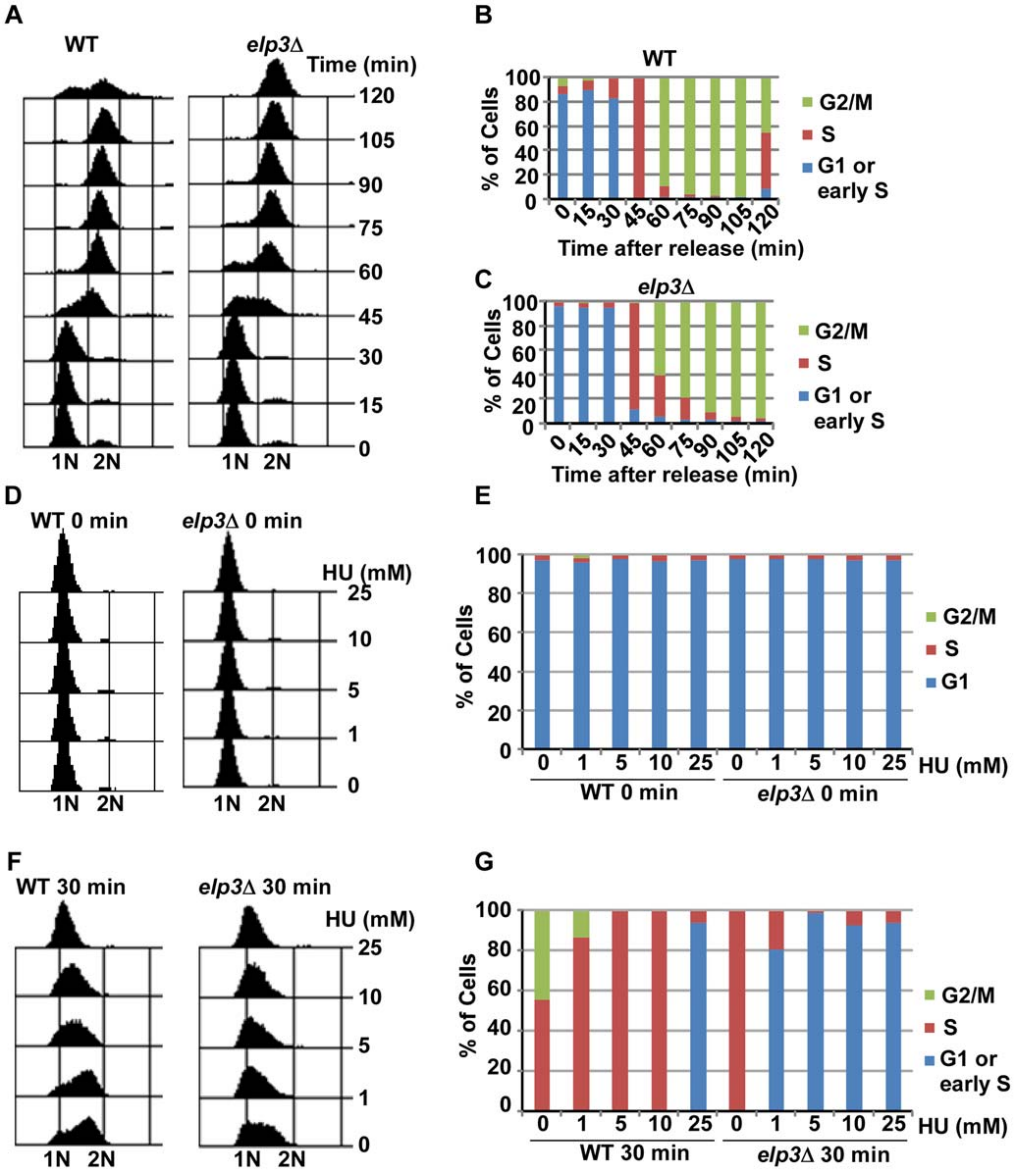
Effect of loss of Elp3 on S-phase progression in the absence and presence of HU. (A–C) The *elp3*Δ mutant cells show slightly delayed in progression through S phase. Wild-type (WT) and *elp3*Δ mutant cells synchronized at G1 with a factor were released into fresh media at 25°C. Every 15 min after release, cells were collected for analysis of DNA content using flow cytometry. Flow cytometry profiles (A) as well as quantification of the percentage of cells in different cell cycle stage for wild type (B) and *elp3*Δ mutant cell (C) are shown. (D–G) *ELP3* depletion is slows or prevents progression through S phase in the presence of low concentrations of Hydroxyurea. Wild-type (WT) and *elp3*Δ mutant cells were arrested in G1 with α factor and released into YPD media containing different concentrations of HU as indicated. Samples were harvested at 0 min in HU (D,E) or 30 min in HU (F,G) for analysis of DNA content by flow cytometry. Flow cytometry profiles (D,F) and quantification of percentage of cells at different cell cycle stage (E,G) were shown.

To better understand the HU sensitivity of the *elp3*Δ mutant cells, we analyzed how the *elp3*Δ mutant cells progressed through the cell cycle in the presence or absence of HU. First, exponentially growing wild type (WT) and *elp3*Δ mutant cells were arrested into G1 phase using á factor and then released into fresh media at 25°C. Every 15 mins after release, the cells were collected and DNA content was analyzed by flow cytometry. The *elp3*Δ mutant cells exhibited a slightly prolonged progression through S phase compared to wild type cells (Figure 4A and 4B). Next, we analyzed how wild type and the *elp3*Δ mutant cells progressed through S phase in the presence of low concentrations of HU. Wild type and *elp3*Δ mutant cells synchronized in G1 using α factor were released into fresh media with or without the indicated concentration of HU at 30°C. Both wild type and *elp3*Δ mutant cells were arrested in G1 phase by á factor (Figure 4D and 4E). Wild type cells progressed through S phase normally in the presence of 1 mM HU compared to those in the absence of HU (Figure 4F and 4G). In contrast, most of the *elp3*Δ mutant cells were arrested at G1 phase or early S phase at this HU concentration. The difference in progression through S phase between wild type and the *elp3*Δ mutant cells was also observed in the presence of higher concentrations of HU (5 and 10 mM). However, the difference in cell cycle progression between wild type and *elp3*Δ mutant cells was not detectable in the presence of 25 mM HU. This is most likely due to the fact that wild type cells were blocked at early S phase in the presence 25 mM HU (Figure 4F and 4G) and that flow cytometry analysis of DNA content could not differentiate cells in G1 phase from those in early S phase. Together, our data suggest that Elp3 is needed for efficient progression through S phase under normal growth conditions and is essential when HU-induced replication stress occurs.

### Elp3 maintains genome stability

**Figure 5:**
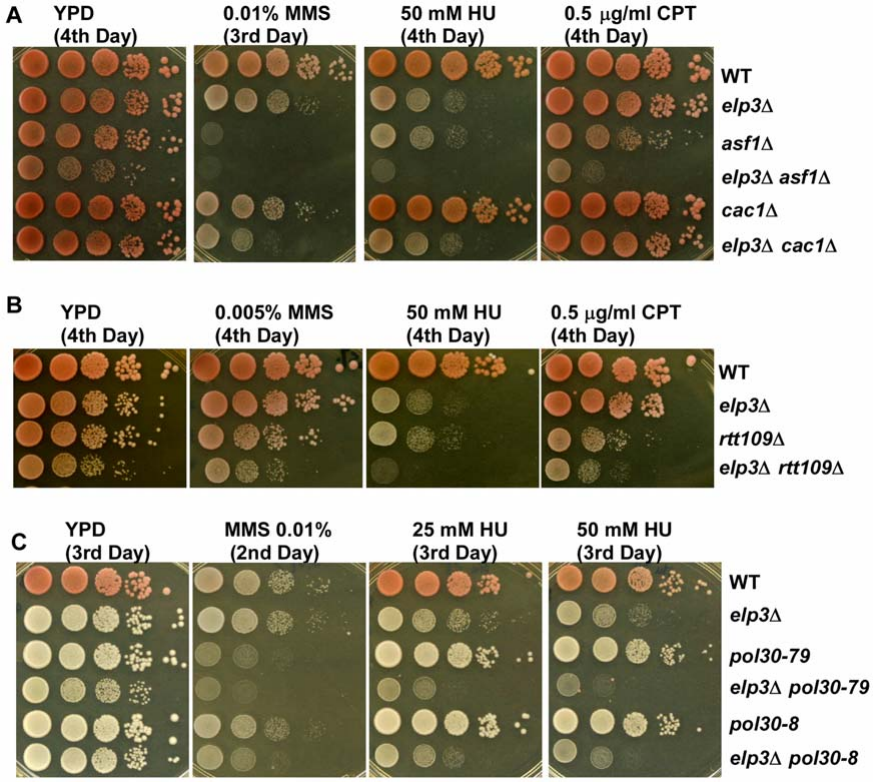
Elp3 is important to resist DNA damaging agents in the absence of Asf1 and Rtt109, two proteins that are essential for acetylation of lysine 56 of H3 in cells. (A) Elp3 functions in a pathway that is genetically distinct from Asf1 in response to insults from DNA damaging agents. Ten-fold serial dilutions of wild type, *asf1*Δ, *cac1*Δ and *elp3*Δ single, or *asf1*Δ *elp3*Δ and *cac1*Δ *elp3*Δ double mutant cells were plated on non-selective YPD medium or YPD medium containing indicated concentrations of DNA damaging agents MMS, HU and CPT. A more extensive analysis of the data is presented in Figure S2. (B) The *elp3*Δ *rtt109*Δ cells are as sensitive towards DNA damaging agents as *elp3*Δ *asf1*Δ mutant cells. The experiment was performed as described in A. A more extensive analysis of the data is presented in Figure S3. (C) The *elp3*Δ mutant cells exhibit distinct sensitivity towards DNA damaging agents MMS and HU when combined with two different PCNA mutant alleles. The experiment was performed as described in A using strains with relevant genotype indicated at the right. A more extensive analysis of the data is presented in Figure S4. Pictures were taken after incubation at 30°C for indicated amounts of time.

In a search for histone acetyltransferases that exhibited synthetic phenotypes with Asf1, we made a series of double mutant strains containing mutations in *ASF1* and each of the genes encoding lysine acetyltransferases and determined how each double mutant cells responded to the insult of DNA damaging agents. Mutations in Elp3 and Gcn5 exhibited synthetically slow growth defects with the *asf1*Δ mutant cells, whereas mutations in other non-essential acetyltransferases including Hap1, Hap2, Hat1 and Sas3 had no apparent additional growth defects in the absence of Asf1 (this report on Elp3 and R.J. Burgess, H. Zhou, J. Han and Z. Zhang, submitted for publication for Gcn5). To confirm these findings, we analyzed how *elp3*Δ *asf1*Δ double mutant cells grew under normal conditions and responded to DNA damaging agents such as HU and MMS. As reported, the *asf1*Δ mutant cells were very sensitive to three DNA damaging agents MMS, HU and CPT [23]. The *elp3*Δ mutant cells were partially sensitive to 0.01% MMS and were not sensitive to CPT (Figure 5A and Figure S2). On the other hand, the *elp3*Δ mutant cells were as sensitive to HU as the *asf1*Δ mutant cells (Figure 5A and Figure S2). Moreover, the *elp3*Δ *asf1*Δ mutant cells grew slower than either mutant alone, even on normal growth media such as YPD, and were more sensitive to all these DNA damaging agents than either *elp3*Δ or *asf1*Δ single mutant alone (Figure 5A and Figure S2). By contrast, the *elp3*Δ *asf1*Δ cells expressed the GFP reporter gene at the *HMR* locus at the same level as *asf1*Δ cells using a semi-quantitative silencing assay (Table S1).

Recent studies have shown that Asf1 functions in the same genetic pathway as Rtt109 to maintain genome stability[34]–[37],[58].Rtt109 is the lysine acetyltransferase that acetylates lysine 56 of H3 (H3K56Ac). We, therefore, tested whether the *elp3*Δ mutation also exhibited synthetic phenotypes with the *rtt109*Δ mutation. Like the *elp3*Δ *asf1*Δ mutant cells, the *elp3*Δ *rtt109*Δ mutant cells grew more slowly than either single mutant alone and were much more sensitive to HU and MMS than either *elp3*Δ or *rtt109*Δ single mutant alone, suggesting that the synthetic phenotypes observed in *elp3*Δ *asf1*Δ mutant cells may be due to loss of H3K56Ac in the *elp3*Δ cells (Figure 5B and Figure S3). These combined results with HU and MMS suggest that Elp3 functions in a pathway independent of Asf1.

CAF-1 is another histone chaperone that functions with Asf1 in DNA replication-coupled nucleosome assembly. Therefore, we tested whether *ELP3* also genetically interacted with *CAC1*, encoding the large subunit of CAF-1. In contrast to deletion of *ASF1*, deletion of *CAC1* did not alter cell viability in the presence of the *elp3*Δ mutation (Figure 5A) under normal growth conditions as well as in the presence of HU (Figure S1). Deletion of *CAC1* did not cause sensitivity to HU, but *cac1*Δ cells were slightly sensitive to 0.01% MMS and the *cac1*Δ *elp3*Δ mutants were more sensitive than cells containing either mutant alone. Thus, there was a synthetic effect with MMS between mutation in *CAC1* and *ELP3*.

In both yeast and human cells, PCNA recruits CAF-1 to replicating DNA through physical interactions [19],[20],[43]. We have shown that two PCNA mutant alleles, *pol30-79* and *pol30-*8, affected silencing and DNA damage sensitivity differently [43]. The *pol30-8* mutant allele disrupts the CAF-1 dependent pathway, whereas *pol30-79* affects silencing and DNA damage sensitivity through a CAF-1 independent pathway [43]. As shown in Figure 5C and Figure S4, the *elp3*Δ *pol30-79* double mutant cells were much more sensitive to DNA damaging agents MMS and HU than either *pol30-79* and *elp3*Δ single mutant cells alone. By contrast, the *elp3*Δ *pol30-8* double mutant cells displayed synthetic defects in the presence of MMS, but not in the presence of HU compared to *elp3*Δ or *pol30-8* mutant alone. This is consistent with the fact that the *cac1*Δ *elp3*Δ double mutant cells also displayed synthetic defects in the presence of MMS, but not HU. Thus, ELP3 genetically interacts with three components (CAF-1, Asf1 and PCNA) of replication- and repair-coupled nucleosome assembly.

### Elp3 genetically interacts with histones H3 and H4 implicated in nucleosome assembly

**Figure 6:**
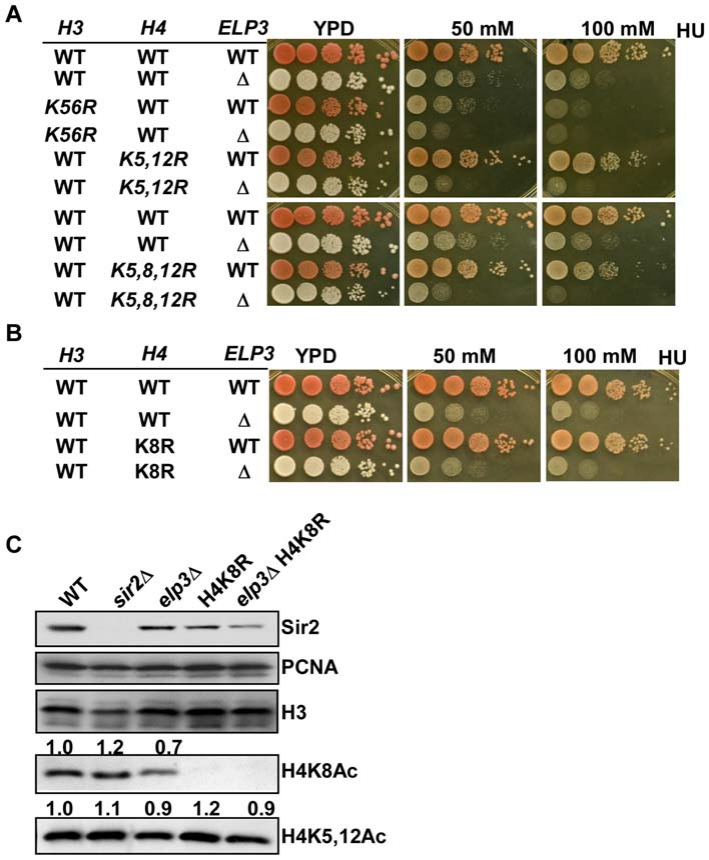
The *elp3*Δ mutation genetically interacts with mutations on lysine residues of H3 and H4 that have been implicated in replication-coupled nucleosome assembly. (A) The *elp3*Δ mutant cells are much more sensitive to HU when combined with lysine-to-arginine mutations at lysine 56 of H3 or at lysine residues 5, 8, and 12 of H4. The experiments were performed as described in Figure 5. Images for HU sensitivity were taken after incubation at 30°C for four days. (B) The *elp3*Δ mutant cells do not exhibit additional phenotype when combined with a lysine-to-arginine mutation at lysine 8 of H4. (C) The level of acetylation of H4 lysine 8 is reduced in the *elp3*Δ mutant cells. Cell extracts were prepared from wild type and different mutant cells and analyzed by Western blot using antibodies against indicated proteins. The band intensity was quantified using Quantity One (Bio-Rad laboratories).

We and others have shown that H3K56Ac promotes efficient DNA replication-coupled nucleosome assembly [28],[29]. Moreover, in addition to H3K56Ac, acetylation of lysine residues 5, 8 and 12 of H4 have been implicated in PCNA and CAF-1-dependent nucleosome assembly [46]. We, therefore, analyzed the phenotypes of the *elp3*Δ mutant when combined with a H3K56R mutant, in which H3 lysine 56 was replaced with arginine, as well as H4 mutants with mutations at lysine 5, 8 and 12. As shown in Figure 6A, the *elp3*Δ *H3K56R* double mutant cells were more sensitive to the DNA damaging agent HU than either *elp3*Δ or *H3K56R* single mutant alone, supporting the idea that the DNA damage sensitivity observed in *asf1*Δ *elp3*Δ mutant cells is due to a loss of H3K56Ac in the *elp3*Δ mutant cells. Moreover, we also observed that double mutant cells containing the *elp3*Δ and *H4K5,12R* mutations were much more sensitive to HU than either *elp3*Δ cells or cells expressing *H4K5,12R* mutations alone. These results, taken together with the HU results above, suggest that Elp3 genetically interacts with several components (Asf1, Rtt109, H3K56Ac and H4K5, 12Ac) that are known to be involved in DNA replication-coupled nucleosome assembly.

### Elp3 is one of the lysine acetyltransferases that acetylates H4 lysine 8

*In vitro*, H4 lysine 8 is one of the preferred acetylation sites for the Elongator complex [9]. We observed that mutation at lysine 8 of H4 did not exacerbate the HU sensitivity of *elp3*Δ *H4K5,12R* double mutant cells (Figure 6A). Mutation of the H4K8 lysine to arginine did not cause HU sensitivity and the *elp3*Δ *H4K8R* double mutant cells exhibited the same degree of HU sensitivity as the *elp3*Δ mutant cells (Figure 6B). To determine whether Elp3 is essential for acetylation of H4 lysine 8 (H4K8Ac), we analyzed the level of H4K8Ac in wild type and *elp3*Δ mutant cells by Western blot. As a negative control, we included the H4K8R mutant strains. As shown in Figure 6C, the H4K8Ac level was reduced in the *elp3*Δ mutant cells to 70% of the level found in wild type cells, suggesting that Elp3 is one, but not the only lysine acetyltransferase that targets H4K8. Because Sir2 is known to deacetylate lysine 16 of H4, we also analyzed H4K8 level in *sir2*Δ mutant cells and found that H4K8Ac did not change the level of acetylation. Together, these results suggest that Elp3 is one of the lysine acetyltransferases that acetylates H4K8Ac but that the H4K8 acetylation did not phenocopy mutations in *ELP3*.

### PCNA interacts with the Elongator complex

**Figure 7:**
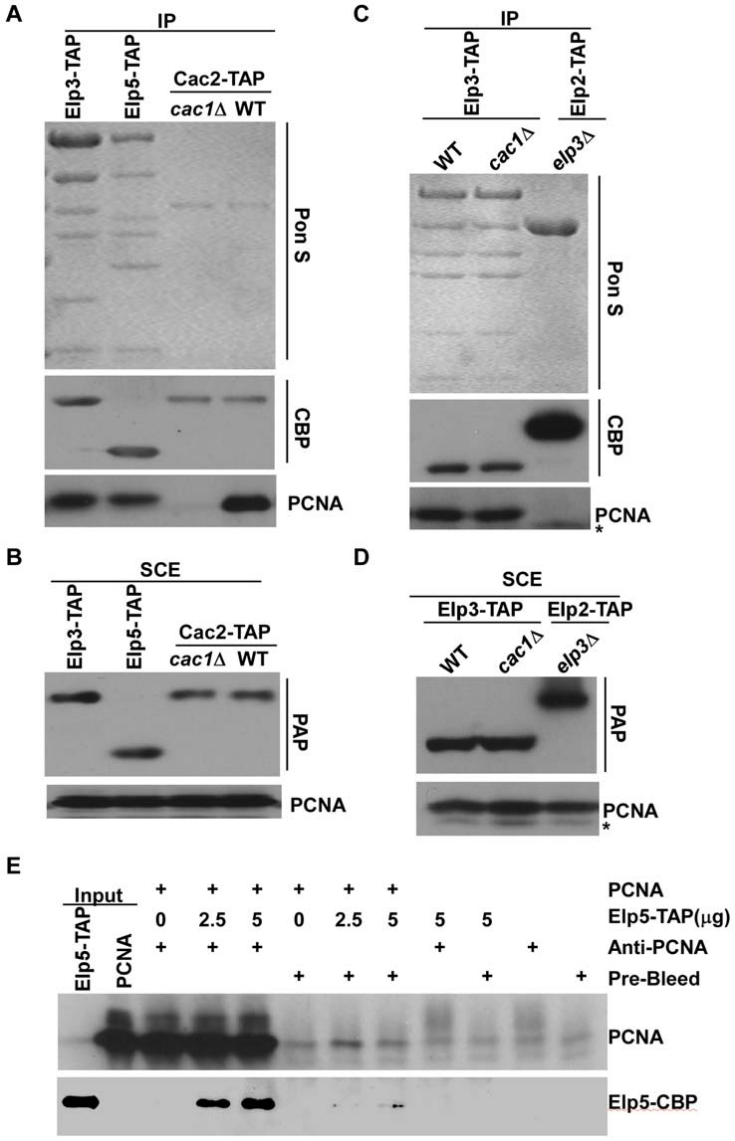
PCNA is associated with the Elp3 complex. (A,B) PCNA co-purified with Elp3 complex in yeast cells. Elp3, Elp5 (two subunits of the Elongator complex), and Cac2-TAP (a subunit of CAF-1 complex) were purified from equal amounts of yeast cells. Co-purified protein were detected by Ponceau S staining or by Western blotting using antibodies against calmodulin binding peptide (CBP), which is present on each TAP tag protein after TEV cleavage, and PCNA. We used a yeast strain (*Cac2-TAP cac1Δ*) as a negative control as the interaction between PCNA and CAF-1 is mediated mainly through Cac1. As a control, we also analyzed proteins in soluble cell extracts (SCE) using antibodies against PCNA and IgG (PAP) (B). (C) The association of PCNA with the Elongator complex requires an intact complex. Elp3-TAP proteins were purified from wild type or *cac1*Δ mutant cells, or Elp2-TAP were purified from the *elp3*Δ mutant cells and co-purified proteins were detected by Ponceau S staining or by Western blotting. (D) As a control, proteins in soluble cell extracts (SCE) were analyzed by Western blot using antibodies against PAP and PCNA. (E) The Elp3 complex binds PCNA *in vitro*. Recombinant PCNA molecules (2.5 µg) were incubated with indicated amounts of Elp3 complex purified using Elp5-TAP. The mixtures were precipitated using anti-PCNA serum and immunoprecipitated proteins were detected by Western blot using antibodies against PCNA and CBP. Pre-bleed sera were used as a negative control.

Next, we tested for biochemical interactions between Elp3 and PCNA or its chromatin assembly binding partner CAF-1. The Elp3 containing complex did not exhibit any acetylation activity towards CAF-1 or PCNA *in vitro* (data not shown). Therefore, we tested whether the Elp3 containing complex physically interacted with CAF-1 and PCNA. Briefly, the Elongator complex was purified from yeast cells using Elp3-TAP and Elp5-TAP. As controls, we also purified CAF-1 complex using Cac2-TAP from wild type and *cac1*Δ mutant cells. Cac1 was not detected in the purified Elp3-TAP or Elp5-TAP complexes, while under the same conditions, Cac1 co-purified with Cac2-TAP from wild type cells (data not shown). In agreement with previous observations, PCNA co-purified with Cac2-TAP from wild type cells, but not with Cac2-TAP from the *cac1*Δ mutant cells [29],[43],[47]. Interestingly, PCNA also co-purified with both Elp3-TAP and Elp5-TAP (Figure 7A and 7B), suggesting that PCNA interacts with the Elongator complex.

Because PCNA is known to interact with CAF-1 through Cac1 [43] (Figure 7A and 7B), it is entirely possible that the interaction between PCNA and the Elongator complex is mediated through CAF-1. To test this possibility, we purified Elp3 from *cac1*Δ mutant cells and found that PCNA still co-purified with Elp3. Thus, the interaction between PCNA and the Elongator complex is not mediated through CAF-1. Moreover, PCNA was not detectable in the Elp2-TAP purification from *elp3*Δ mutant cells (Figure 7C and 7D). This result suggests that the interaction between PCNA and the Elongator complex is specific and that Elp2 is not likely to be the primary determinant for the Elongator complex to bind PCNA.

None of the six subunits of the Elongator complex contain an obvious PCNA interaction motif found in other proteins [59]. To ascertain whether the Elongator complex binds to PCNA directly, we tested whether PCNA interacted with the Elongator complex *in vitro*. PCNA was expressed and purified from *E. coli* and was incubated with the Elongator complex purified from yeast cells using Elp5-TAP. The reaction mixtures were then immunoprecipitated using antibodies against PCNA. As shown in Figure 7E, Elp5 was immunoprecipitated with PCNA *in vitro* only when antibodies against PCNA were used and only when PCNA was present. Thus, PCNA interacts directly with the Elongator complex *in vivo* and *in vitro*.

## Discussion

In this report, we have shown that the *elp3*Δ mutant cells partially lose gene silencing of the *URA3* reporter gene at a telomere (chromosome VIIL) and the *ADE2* reporter gene, but not the *GFP* reporter gene at the *HMR* locus, and are sensitive to DNA damaging agents HU and MMS. A similar degree of silencing defects and sensitivity towards DNA damaging agents were observed when other Elongator subunit genes were deleted. The results suggest that an intact Elongator complex is required for efficient transcriptional silencing and an optimal DNA damage response. Supporting this idea, we have shown that mutations within the radical SAM domain of Elp3 compromise formation of the Elongator complex and exhibit the same phenotypes as found for the *elp3*Δ mutant cells. Moreover, the *elp3*Δ mutant enhances the HU and MMS DNA damage sensitivity of *asf1*Δ and *rtt109*Δ deletions and the *H3K56R* mutation and enhances the sensitivity of *cac1*Δ mutant in the presence of MMS. We suggest, therefore, that Elongator functions in a pathway in parallel to the *CAF-1* and *ASF1* chromatin assembly processes.

The Elongator complex interacted directly with PCNA, a protein that also interacts with CAF-1 and facilitates the link between nucleosome assembly and DNA synthesis, either in S phase or during DNA repair. However, we could not detect a single complex containing CAF-1, PCNA and Elongator, suggesting that Elongator and PCNA form a separate complex that functions independently from the CAF-1-PCNA complex. This conclusion is supported by genetic data that shows that the *pol30-8* mutation, which is defective in binding CAF-1 [43], displays synthetic defects when combined with *elp3*Δ in the presence of MMS. Together, these results reveal a new function for Elp3 in maintaining genome stability and suggest that Elp3 functions in this process through a pathway genetically and physically linked to PCNA.

We reported here that the *elp3*Δ mutant cells are highly sensitive to HU, an inhibitor of ribonucleotide reductase (RNR) that reduces dNTP pools and thereby prevents the firing of late replication origins and slows down DNA synthesis from early origins [60],[61]. HU also activates the checkpoint kinase Rad53, which in turn leads to induction of RNR gene expression. Because the Elongator complex is best known for its function in transcription elongation, it is possible that HU sensitivity of the *elp3*Δ mutant cells is due to altered expression of *RNR* genes or other genes involved in the checkpoint response, however, microarray analysis of mRNA levels indicate that the expression of *RNR* genes is not affected in the *elp3*Δ mutant cells [8]. Moreover, the expression of *RNR1* and *RNR3* genes was induced by HU in the *elp3*Δ mutant cells to a similar level as that of wild type cells (data not shown). These results suggest that the HU sensitivity of the *elp3*Δ mutant cells is likely not due to defects in a transcriptional response to checkpoint activation. Instead, we suggest that sensitivity towards HU and other DNA damaging agents including MMS displayed by the *elp3*Δ mutant cells arise from a defect in a process that involves PCNA and cooperates with ASF1 and CAF-1 dependent DNA replication- or DNA repair-coupled nucleosome assembly. Supporting this idea, we have shown that the *elp3*Δ mutation genetically interacts with mutations at lysine 56 of H3 as well as lysine residues 5 and 12 of histone H4 in the response to HU. Moreover, low concentrations of HU slow down S phase progression of the *elp3*Δ mutant cells significantly more than wild type cells. Lastly, genome wide studies reveal that the *elp3* mutant exhibits a synthetically lethal/slow growth phenotype with a mutation in *RFC4* [58]. Rfc4 is a subunit of the RFC clamp-loader complex that loads the PCNA DNA polymerase clamp onto DNA during DNA replication and repair [21].

There are three possible models of how Elp3 is involved in regulating DNA replication- or DNA repair-coupled nucleosome assembly. First, it is possible that the defects displayed by the *elp3*Δ mutant are due to altered expression of genes such as PCNA, CAF-1 and Asf1 involved in nucleosome assembly. This is not likely since microarray analysis indicates the expression of the genes known to be involved in DNA replication-coupled nucleosome assembly pathway are not affected in cells without Elongator complex [8]. Second, the Elongator complex may acetylate newly synthesized histones before deposition onto replicating DNA. Supporting this idea, the Elongator complex is mostly localized in the cytosol and one of the preferential lysine residues acetylated by the Elongator complex is lysine 8 of histone H4 [9]. Epistasis analysis indicates that the *elp3*Δ mutation when combined with mutations at lysine 8 of H4 exhibited a phenotype similar to the phenotype observed for the *elp3*Δ mutation and the level of H4K8Ac was partially reduced in the *elp3*Δ mutant cells. Curiously, acetylation of lysine residues 5, 8 and 12 of H4 is present on H4 co-purified with CAF-1 from both yeast and mammalian cells [46],[47]. Thus, it is possible that the Elp3 containing complex may be one of the enzymes that acetylates lysine 8 of H4 and that this modified histone ends up complexed with CAF-1 for deposition during DNA synthesis. However, we noticed that cells lacking Elp3 were sensitive to HU and mutations at lysine 8 of H4 did not show increased HU sensitivity compared to wild type cells, suggesting that the HU sensitivity of the Elp3 mutant cells is not likely due to a loss of acetylation of lysine 8 of H4.

A third possible model derives from our observation that PCNA interacts with the Elongator complex, implying a nuclear function for Elongator complex that is coupled to DNA synthesis and nucleosome assembly. It is possible that the Elongator complex travels with PCNA during DNA replication to modulate chromatin structure and/or proteins involved in DNA replication to ensure that DNA synthesis is coupled to nucleosome assembly during S phase of the cell cycle. The finding that deletion of both *GCN5* and *ELP3* is synthetically lethal and that the lethality can be suppressed by deletion of either *SIR1*, *SIR3* or *SIR4* suggests that in the absence of the two acetyltransferases, cellular chromatin becomes hypo-acetylated and Sir proteins spread to euchromatin and inhibit genes that are normally not silenced [12]. We suggest that the lethality in *elp3Δ* and *gcn5Δ* strains is due to inappropriate assembly of normal chromatin during DNA replication that leads to secondary effects that cause lethality, such as gene expression defects. In the absence of the Elongator complex alone, some hypo-acetylation of histones likely occurs, thereby compromising chromatin assembly, but this is not sufficient to kill the cells. The defect in *elp3Δ* cells is only revealed when DNA damage is induced, suggesting that DNA damage creates a condition where the Elongator mediated function related to chromatin assembly is more important, possibly as a result of its interaction with PCNA. This model is consistent with our observation that in the absence of Elongator and Asf1 or Rtt109, the chromatin assembly defect is enhanced, even in cells where DNA damage is not externally induced (Figure 5). Moreover, simultaneous depletion of Elongator and CAF-1 does not compromise the growth of cells, but in the presence of 0.01% MMS, *elp3*Δ *cac1*Δ mutant cells display synthetic defects. Thus, redundancy of chromatin assembly mechanisms and acetyltransferases ensures that robust chromatin assembly occurs during S phase and during DNA repair.

In addition to the Elongator complex, three other histone acetyltransferases are known to interact with factors involved in DNA replication-coupled nucleosome assembly. First, Sas2 has been shown to interact with Asf1 and CAF-1 [49],[50]. Sas2 forms a complex with Sas4 and Sas5, acetylates predominantly lysine 16 of H4 and functions in transcriptional silencing [51],[52],[62],[63]. However, the functional significance of this interaction remains unclear as it appears that acetylation of lysine 16 of H4 at telomeric heterochromatin is normal in cells lacking CAF-1 and Asf1 [64]. Second, it has been shown recently that Hat1 binds to Asf1 in yeast cells [48]. Hat1 forms a complex with Hat2 and Hif1 and acetylates lysine 5 and 12 of H4 in yeast and mammalian cells [65]–[67]. Third, Asf1 regulates acetylation of H3 lysine 56 by presenting H3–H4 to Rtt109-Vps75 complex [31],[34],[58] thereby directly regulating the binding of H3 with two other histone chaperones CAF-1 and Rtt106 and subsequent nucleosome assembly during S phase of the cell cycle [29]. Thus, there are multiple histone acetyltransferases that function directly or indirectly during the DNA replication-coupled nucleosome assembly process in budding yeast. Why then are several histone acetyltransferases involved in this process? It is known that acetylation of H3 lysine 56 by Rtt109 and H4 lysine 5 and 12 are present on newly-synthesized histones [68],[69]. Thus, acetylation of these lysine residues is likely to facilitate deposition of new H3–H4 onto replicating DNA for *de novo* nucleosome assembly. In addition to new H3–H4, parental histones must be transferred to replicating DNA during S phase, a process that is poorly understood. It has been suggested recently that Asf1 can disrupt parental nucleosomes following DNA replication in human cells [70]. It is possible some of these HATs are also involved in acetylation of parental histones to facilitate nucleosome remodeling and subsequent transfer of parental histones onto replicating DNA. Supporting this idea, it has been shown that PCNA interacts with lysine acetyltransferase p300 in human cells [71]. It is proposed that the PCNA-p300 interaction facilitates chromatin remodeling at the DNA damage sites by p300 [71]. Thus, it is possible that PCNA may also recruit the Elongator complex to modulate chromatin structure during DNA replication or in response to DNA damage.

The acetylation patterns of new-synthesized H4 is conserved from yeast to human cells [69]. Recently, it has been shown that H3K56Ac is also present in mammalian cells [72]–[75]. Moreover, H3K56Ac likely has a function in replication-coupled nucleosome assembly and maintenance of genome stability [72],[74]. Thus, replication-coupled nucleosome assembly in higher eukaryotic cells is also likely regulated by multiple acetylation events catalyzed by different HATs. The future challenge will be to determine whether other histone acetyltransferases and/or other histone modifying enzymes also play a role in the regulation of the DNA replication-coupled nucleosome assembly process and study how these histone modifications are coupled to chromatin assembly at the DNA replication fork and how they influence the inheritance of states of gene expression and maintenance of genome stability in yeast and mammalian cells.

## Materials and Methods

### Ethics statement

This study was conducted according to the principles expressed in the Declaration of Helsinki.

### Yeast strains

All yeast strains used in this study were derived from the parental W303 background (*leu2-3,112, ura3-1, his3-11, trp1-1, ade2-1, can 1-100*) and were listed in Table S2. Standard yeast media and manipulations were used to generate all the yeast strains. Genes encoding putative histone/protein acetyltransferases were deleted from the yeast using homologous recombination. Standard cloning and site-directed mutagenesis procedures were followed to generate plasmids used in the study.

### Yeast gene silencing assays and assays for the sensitivity towards DNA damaging agents

To assay telomeric silencing, 10-fold serial dilutions of freshly grown yeast cells were spotted onto non-selective YPD media to assay cell viability and synthetic media lacking uracil or media containing the drug FOA to assay silencing. To analyze the sensitivity of cells to different DNA damaging agents including hydroxyurea (HU) and methyl methane sulfonate (MMS), 10-fold serial dilutions of yeast cells were spotted onto YPD plates containing different concentrations of hydroxyurea (HU, 25 mM, 50 mM, 100 mM, and 150 mM), methyl methanesulfonate (MMS, 0.001%, 0.003% and 0.01%), or camptothecin (CPT, 0, 1, 2.5 and 5 µg/ml). These plates were incubated at 30°C for at least two days before being photographed using a digital camera (Cannon). In most figures, results from one representative drug concentration were shown and the entire results were shown in Figures S1, S2, S3, S4.

### Purification of the Elp3 complex using Tandem affinity purification

The experiment was performed essentially the same as described [29]. Briefly, exponentially growing yeast cells were harvested by centrifugation and washed once with 10% glycerol. The volume of yeast cells was estimated and an equal volume of buffer A (25 mM Tris, pH 8.0, 100 mM NaCl, 1 mM EDTA, 10 mM MgCl_2_, 0.01% NP40, 1 mM DTT) with protease inhibitors (l mM PMSF, 1 mM Benzamidine, 1 mM Pefablock) and 15 KU/ml DNase I was used to re-suspend yeast cells, which were frozen in liquid nitrogen. The frozen yeast cells were ground with dry ice in a coffee grinder. Cell lysates were cleared by centrifugation and incubated with IgG sepharose beads (GE Healthcare) for 2 hrs. After the beads were washed extensively, the proteins were eluted by digestion with the TEV protease. The eluted proteins were then bound to calmodulin beads. Proteins bound to calmodulin beads were either eluted in a buffer containing EGTA for activity analysis and *in vitro* binding with PCNA or with SDS sample buffer for analysis by Western blot or silver staining.

### Purification of PCNA

The PCNA protein was purified as described [48] with minor modifications. Briefly, bacterial cells [BL21(DE3)] transformed with plasmid for PCNA expression were induced with 0.5 mM isopropylthiogalactoside (IPTG) for 3 hrs. Cell extracts were prepared by passing through a french press in buffer A (25 mM Tris, pH 7.4, 25 mM NaCl, 1 mM EDTA, 0.01% NP-40, 1 mM DTT and 5 mM NaHSO_3_, 0.2 mM PMSF, 2 µg/ml of pepstatin A and 2 mM benzamidine HCl). The extracts were cleared by centrifugation. The resulting supernatants were applied to a Q-Sepharose column (GE Healthcare) after adjusting the salt concentration to 0.2 M NaCl. PCNA proteins were eluted with a gradient of 0.2–0.7 M NaCl in buffer A. The eluted PCNA proteins were collected and dialyzed in HAP equilibrium buffer (25 mM K_2_HPO_4_, pH 7.0, 0.01% NP-40, 10% glycerol, 0.2 mM PMSF, 1 mM DTT, 0.6 µg/ml pepstatin A and 2 mM benzamidine HCl). The flow through was then loaded onto the pre-equilibrium HAP (Hydroxyapatite HTP gel, BioRad) column and washed with 5 loading volumes of HAP equilibrium buffer, 5 volumes of equilibrium buffer with 62.5 mM containing K_2_HPO_4_ , and eluted with 2 loading volumes of equilibrium buffer containing 400 mM K_2_HPO_4_. Fractions containing PCNA were then pooled and purified further by using the HiLoad 16/60 Superdex 200 gel-filtration column. The purified PCNA was dialyzed against buffer B (25 mM Tris, pH 7.4, 200 mM NaCl, 1 mM EDTA, 0.01% NP-40, 10% glycerol and 1 mM DTT) and stored at −80°C before use.

### Interaction between PCNA and the Elp3 complex *in vitro*

Elp3 complex was purified from yeast cells by affinity chromatography with Elp5-TAP as described above. To test whether PCNA bound to the Elp3 complex, 2.5 µg of purified PCNA was incubated with different amounts of Elp3 complex in TBS buffer containing 0.01% NP-40 at 4°C for 2 hrs. Then 5 µg polyclonal antibodies against PCNA were added to the reaction mixtures and incubated at 4°C overnight. Pre-washed Protein G Sepharose (GE Healthcare) were added to the mixture and incubated at 4°C for 2 hrs. After the beads were washed extensively, the bound proteins were eluted by SDS-PAGE loading buffer, resolved by 10% SDS-PAGE and detected by Western blotting with antibodies against PCNA or calmodulin binding peptide (CBP) that was fused at the C-terminus of Elp5 after TEV cleavage of the TAP tag. Pre-bleed sera were used as a negative control for immunoprecipitation.

### Quantification of flow cytometry scan

Yeast cells were grown to log phase and analyzed DNA content by flow cytometry. Quantification of different cell phase was performed by ModFit LT ™ for MacV 3.0.

## Supporting Information

Figure S1 The *ELP3* complex is important for resistance to DNA damaging agents. Ten fold series dilution of wild type or *elp3*Δ mutant cells were spotted onto medium containing different concentration of hydroxyurea (HU, A), methyl methane sulfate (MMS, B) or camptothecin (CPT, C). (D) Other subunits of the holo-elongator complex are required for the resistance to DNA damaging agents. Ten-fold serial dilutions of wild type or mutants lacking each of the five subunits of the holo-elongator complex were plated on medium containing different concentrations of bleomycin. The plates were incubated at 30°C for three days before pictures were taken.
(1.74 MB TIF)

Figure S2 The *elp3*Δ mutation exhibits synthetic genetic interactions with *asf1*Δ but not *cac1*Δ mutant in response to DNA damaging agents. Ten-fold serial dilutions of yeast cells with relevant genotype identified at the right were spotted onto medium containing different HU (A), MMS (B) and CPT (C). Pictures were taken after three days of incubation at 30°C.
(4.07 MB TIF)

Figure S3 The *elp3*Δ *rtt109*Δ double mutant cells exhibit similar phenotypes to those of *elp3*Δ *asf1*Δ double mutant cells. Ten fold serial dilution of yeast cells with relevant genotype shown at the right were assayed for their sensitivity towards HU (A), MMS (B) or CPT (C). Images for cells growing on YPD plates were taken after incubation at 30°C for four days except that those indicated.
(2.52 MB TIF)

Figure S4 The *elp3*Δ mutant cells are more sensitive to DNA damage agents when combined with a PCNA mutant allele. Ten-fold serial dilution of yeast cells with relevant genotype shown at the right were assayed for their sensitivity towards MMS (A) or HU (B). Images for cells growing on YPD plates were taken after incubation at 30°C for three days.
(2.35 MB TIF)

Table S1 The *elp3*Δ mutant does not exhibit synergistic loss of silencing at *HMR* locus in the absence of Asf1 using *hmr::GFP* silencing assay. The GFP transgene was integrated at the *HMR* locus in each strain with relevant genotype listed in the first column. Expression of *GFP* in wild type or various mutants was determined by *FACS*. Percentage of GFP expressing cells of each strain was shown. Because autofluorescence influenced the results significantly when the percentage of GFP positive cells was less than 1%, and we regarded the effect of any strains with less than 1% GFP cells as negligible.
(0.04 MB DOC)

Table S2 Yeast strains used in this study.
(0.09 MB DOC)

## References

[1] Zhang Y, Reinberg D (2001). Transcription regulation by histone methylation: interplay between different covalent modifications of the core histone tails.. Genes Dev.

[2] Strahl BD, Allis CD (2000). The language of covalent histone modifications.. Nature.

[3] Kouzarides T (2007). Chromatin modifications and their function.. Cell.

[4] Groth A, Rocha W, Verreault A, Almouzni G (2007). Chromatin challenges during DNA replication and repair.. Cell.

[5] Li B, Carey M, Workman JL (2007). The role of chromatin during transcription.. Cell.

[6] Wittschieben BO, Otero G, de Bizemont T, Fellows J, Erdjument-Bromage H (1999). A novel histone acetyltransferase is an integral subunit of elongating RNA polymerase II holoenzyme.. Mol Cell.

[7] Frohloff F, Fichtner L, Jablonowski D, Breunig KD, Schaffrath R (2001). Saccharomyces cerevisiae Elongator mutations confer resistance to the Kluyveromyces lactis zymocin.. Embo J.

[8] Krogan NJ, Greenblatt JF (2001). Characterization of a six-subunit holo-elongator complex required for the regulated expression of a group of genes in Saccharomyces cerevisiae.. Mol Cell Biol.

[9] Winkler GS, Kristjuhan A, Erdjument-Bromage H, Tempst P, Svejstrup JQ (2002). Elongator is a histone H3 and H4 acetyltransferase important for normal histone acetylation levels in vivo.. Proc Natl Acad Sci U S A.

[10] Allis CD, Berger SL, Cote J, Dent S, Jenuwien T (2007). New nomenclature for chromatin-modifying enzymes.. Cell.

[11] Paraskevopoulou C, Fairhurst SA, Lowe DJ, Brick P, Onesti S (2006). The Elongator subunit Elp3 contains a Fe4S4 cluster and binds S-adenosylmethionine.. Mol Microbiol.

[12] Kristjuhan A, Wittschieben BO, Walker J, Roberts D, Cairns BR (2003). Spreading of Sir3 protein in cells with severe histone H3 hypoacetylation.. Proc Natl Acad Sci U S A.

[13] Grant PA, Duggan L, Cote J, Roberts SM, Brownell JE (1997). Yeast Gcn5 functions in two multisubunit complexes to acetylate nucleosomal histones: characterization of an Ada complex and the SAGA (Spt/Ada) complex.. Genes Dev.

[14] Huang B, Johansson MJ, Bystrom AS (2005). An early step in wobble uridine tRNA modification requires the Elongator complex.. Rna.

[15] Creppe C, Malinouskaya L, Volvert ML, Gillard M, Close P (2009). Elongator controls the migration and differentiation of cortical neurons through acetylation of alpha-tubulin.. Cell.

[16] Rahl PB, Chen CZ, Collins RN (2005). Elp1p, the yeast homolog of the FD disease syndrome protein, negatively regulates exocytosis independently of transcriptional elongation.. Mol Cell.

[17] Stillman B (1986). Chromatin assembly during SV40 DNA replication in vitro.. Cell.

[18] Smith S, Stillman B (1989). Purification and characterization of CAF-I, a human cell factor required for chromatin assembly during DNA replication in vitro.. Cell.

[19] Moggs JG, Grandi P, Quivy JP, Jonsson ZO, Hubscher U (2000). A CAF-1-PCNA-mediated chromatin assembly pathway triggered by sensing DNA damage.. Mol Cell Biol.

[20] Shibahara K, Stillman B (1999). Replication-dependent marking of DNA by PCNA facilitates CAF-1-coupled inheritance of chromatin.. Cell.

[21] Waga S, Stillman B (1998). The DNA replication fork in eukaryotic cells.. Annu Rev Biochem.

[22] Le S, Davis C, Konopka JB, Sternglanz R (1997). Two new S-phase-specific genes from Saccharomyces cerevisiae.. Yeast.

[23] Tyler JK, Adams CR, Chen SR, Kobayashi R, Kamakaka RT (1999). The RCAF complex mediates chromatin assembly during DNA replication and repair.. Nature.

[24] Mello JA, Sillje HH, Roche DM, Kirschner DB, Nigg EA (2002). Human Asf1 and CAF-1 interact and synergize in a repair-coupled nucleosome assembly pathway.. EMBO Rep.

[25] Groth A, Ray-Gallet D, Quivy JP, Lukas J, Bartek J (2005). Human Asf1 regulates the flow of S phase histones during replicational stress.. Mol Cell.

[26] Tagami H, Ray-Gallet D, Almouzni G, Nakatani Y (2004). Histone H3.1 and H3.3 complexes mediate nucleosome assembly pathways dependent or independent of DNA synthesis.. Cell.

[27] Krawitz DC, Kama T, Kaufman PD (2002). Chromatin assembly factor I mutants defective for PCNA binding require Asf1/Hir proteins for silencing.. Mol Cell Biol.

[28] Chen CC, Carson JJ, Feser J, Tamburini B, Zabaronick S (2008). Acetylated lysine 56 on histone H3 drives chromatin assembly after repair and signals for the completion of repair.. Cell.

[29] Li Q, Zhou H, Wurtele H, Davies B, Horazdovsky B (2008). Acetylation of histone H3 lysine 56 regulates replication-coupled nucleosome assembly.. Cell.

[30] Ozdemir A, Masumoto H, Fitzjohn P, Verreault A, Logie C (2006). Histone H3 lysine 56 acetylation: a new twist in the chromosome cycle.. Cell Cycle.

[31] Han J, Zhou H, Li Z, Xu RM, Zhang Z (2007). H3-K56 acetylation catalyzed by Rtt109 and regulated by Asf1 is required for replisome integrity.. J Biol Chem.

[32] Schneider J, Bajwa P, Johnson FC, Bhaumik SR, Shilatifard A (2006). Rtt109 is required for proper H3K56 acetylation: a chromatin mark associated with the elongating RNA polymerase II.. J Biol Chem.

[33] Tsubota T, Berndsen CE, Erkmann JA, Smith CL, Yang L (2007). Histone H3-K56 acetylation is catalyzed by histone chaperone-dependent complexes.. Mol Cell.

[34] Han J, Zhou H, Li Z, Xu RM, Zhang Z (2007). The Rtt109-Vps75 histone acetyltransferase complex acetylates non-nucleosomal histone H3.. J Biol Chem.

[35] Han J, Zhou H, Horazdovsky B, Zhang K, Xu RM (2007). Rtt109 acetylates histone H3 lysine 56 and functions in DNA replication.. Science.

[36] Recht J, Tsubota T, Tanny JC, Diaz RL, Berger JM (2006). Histone chaperone Asf1 is required for histone H3 lysine 56 acetylation, a modification associated with S phase in mitosis and meiosis.. Proc Natl Acad Sci U S A.

[37] Driscoll R, Hudson A, Jackson SP (2007). Yeast Rtt109 promotes genome stability by acetylating histone H3 on lysine 56.. Science.

[38] Kaufman PD, Kobayashi R, Stillman B (1997). Ultraviolet radiation sensitivity and reduction of telomeric silencing in Saccharomyces cerevisiae cells lacking chromatin assembly factor-I.. Genes Dev.

[39] Enomoto S, Berman J (1998). Chromatin assembly factor I contributes to the maintenance, but not the re-establishment, of silencing at the yeast silent mating loci.. Genes Dev.

[40] Enomoto S, McCune-Zierath PD, Gerami-Nejad M, Sanders MA, Berman J (1997). RLF2, a subunit of yeast chromatin assembly factor-I, is required for telomeric chromatin function in vivo.. Genes Dev.

[41] Tyler JK, Collins KA, Prasad-Sinha J, Amiott E, Bulger M (2001). Interaction between the Drosophila CAF-1 and ASF1 chromatin assembly factors.. Mol Cell Biol.

[42] Linger J, Tyler JK (2005). The yeast histone chaperone chromatin assembly factor 1 protects against double-strand DNA-damaging agents.. Genetics.

[43] Zhang Z, Shibahara K, Stillman B (2000). PCNA connects DNA replication to epigenetic inheritance in yeast.. Nature.

[44] Sharp JA, Fouts ET, Krawitz DC, Kaufman PD (2001). Yeast histone deposition protein Asf1p requires Hir proteins and PCNA for heterochromatic silencing.. Curr Biol.

[45] Celic I, Masumoto H, Griffith WP, Meluh P, Cotter RJ (2006). The sirtuins hst3 and Hst4p preserve genome integrity by controlling histone h3 lysine 56 deacetylation.. Curr Biol.

[46] Verreault A, Kaufman PD, Kobayashi R, Stillman B (1996). Nucleosome assembly by a complex of CAF-1 and acetylated histones H3/H4.. Cell.

[47] Zhou H, Madden BJ, Muddiman DC, Zhang Z (2006). Chromatin assembly factor 1 interacts with histone h3 methylated at lysine 79 in the processes of epigenetic silencing and DNA repair.. Biochemistry.

[48] Fillingham J, Recht J, Silva AC, Suter B, Emili A (2008). Chaperone control of the activity and specificity of the histone H3 acetyltransferase Rtt109.. Mol Cell Biol.

[49] Meijsing SH, Ehrenhofer-Murray AE (2001). The silencing complex SAS-I links histone acetylation to the assembly of repressed chromatin by CAF-I and Asf1 in Saccharomyces cerevisiae.. Genes Dev.

[50] Osada S, Sutton A, Muster N, Brown CE, Yates JR (2001). The yeast SAS (something about silencing) protein complex contains a MYST-type putative acetyltransferase and functions with chromatin assembly factor ASF1.. Genes Dev.

[51] Ehrenhofer-Murray AE, Rivier DH, Rine J (1997). The role of Sas2, an acetyltransferase homologue of Saccharomyces cerevisiae, in silencing and ORC function.. Genetics.

[52] Sutton A, Shia WJ, Band D, Kaufman PD, Osada S (2003). Sas4 and Sas5 are required for the histone acetyltransferase activity of Sas2 in the SAS complex.. J Biol Chem.

[53] Aparicio OM, Billington BL, Gottschling DE (1991). Modifiers of position effect are shared between telomeric and silent mating-type loci in S. cerevisiae.. Cell.

[54] Gottschling DE, Aparicio OM, Billington BL, Zakian VA (1990). Position effect at S. cerevisiae telomeres: reversible repression of Pol II transcription.. Cell.

[55] Sussel L, Vannier D, Shore D (1993). Epigenetic switching of transcriptional states: cis- and trans-acting factors affecting establishment of silencing at the HMR locus in Saccharomyces cerevisiae.. Mol Cell Biol.

[56] Huang S, Zhou H, Katzmann D, Hochstrasser M, Atanasova E (2005). Rtt106p is a histone chaperone involved in heterochromatin-mediated silencing.. Proc Natl Acad Sci U S A.

[57] Petrakis TG, Wittschieben BO, Svejstrup JQ (2004). Molecular architecture, structure-function relationship, and importance of the Elp3 subunit for the RNA binding of holo-elongator.. J Biol Chem.

[58] Collins SR, Miller KM, Maas NL, Roguev A, Fillingham J (2007). Functional dissection of protein complexes involved in yeast chromosome biology using a genetic interaction map.. Nature.

[59] Warbrick E (2000). The puzzle of PCNA's many partners.. Bioessays.

[60] Tercero JA, Diffley JF (2001). Regulation of DNA replication fork progression through damaged DNA by the Mec1/Rad53 checkpoint.. Nature.

[61] Alvino GM, Collingwood D, Murphy JM, Delrow J, Brewer BJ (2007). Replication in hydroxyurea: it's a matter of time.. Mol Cell Biol.

[62] Reifsnyder C, Lowell J, Clarke A, Pillus L (1996). Yeast SAS silencing genes and human genes associated with AML and HIV-1 Tat interactions are homologous with acetyltransferases.. Nat Genet.

[63] Suka N, Luo K, Grunstein M (2002). Sir2p and Sas2p opposingly regulate acetylation of yeast histone H4 lysine16 and spreading of heterochromatin.. Nat Genet.

[64] Huang S, Zhou H, Tarara J, Zhang Z (2007). A novel role for histone chaperones CAF-1 and Rtt106p in heterochromatin silencing.. Embo J.

[65] Kleff S, Andrulis ED, Anderson CW, Sternglanz R (1995). Identification of a gene encoding a yeast histone H4 acetyltransferase.. J Biol Chem.

[66] Ai X, Parthun MR (2004). The nuclear Hat1p/Hat2p complex: a molecular link between type B histone acetyltransferases and chromatin assembly.. Mol Cell.

[67] Verreault A, Kaufman PD, Kobayashi R, Stillman B (1998). Nucleosomal DNA regulates the core-histone-binding subunit of the human Hat1 acetyltransferase.. Curr Biol.

[68] Masumoto H, Hawke D, Kobayashi R, Verreault A (2005). A role for cell-cycle-regulated histone H3 lysine 56 acetylation in the DNA damage response.. Nature.

[69] Sobel RE, Cook RG, Perry CA, Annunziato AT, Allis CD (1995). Conservation of deposition-related acetylation sites in newly synthesized histones H3 and H4.. Proc Natl Acad Sci U S A.

[70] Groth A, Corpet A, Cook AJ, Roche D, Bartek J (2007). Regulation of replication fork progression through histone supply and demand.. Science.

[71] Hasan S, Hassa PO, Imhof R, Hottiger MO (2001). Transcription coactivator p300 binds PCNA and may have a role in DNA repair synthesis.. Nature.

[72] Das C, Lucia MS, Hansen KC, Tyler JK (2009). CBP/p300-mediated acetylation of histone H3 on lysine 56.. Nature.

[73] Xie W, Song C, Young NL, Sperling AS, Xu F (2009). Histone h3 lysine 56 acetylation is linked to the core transcriptional network in human embryonic stem cells.. Mol Cell.

[74] Yuan J, Pu M, Zhang Z, Lou Z (2009). Histone H3-K56 acetylation is important for genomic stability in mammals.. Cell Cycle.

[75] Tjeertes JV, Miller KM, Jackson SP (2009). Screen for DNA-damage-responsive histone modifications identifies H3K9Ac and H3K56Ac in human cells.. EMBO J.

