# Supplementary figures and images for: The Elongator Complex Interacts with PCNA and Modulates Transcriptional Silencing and Sensitivity to DNA Damage Agents
Source: PLoS Genet. 2009 Oct 16;5(10):e1000684. doi: 10.1371/journal.pgen.1000684 (PMC2757915; doi:10.1371/journal.pgen.1000684)

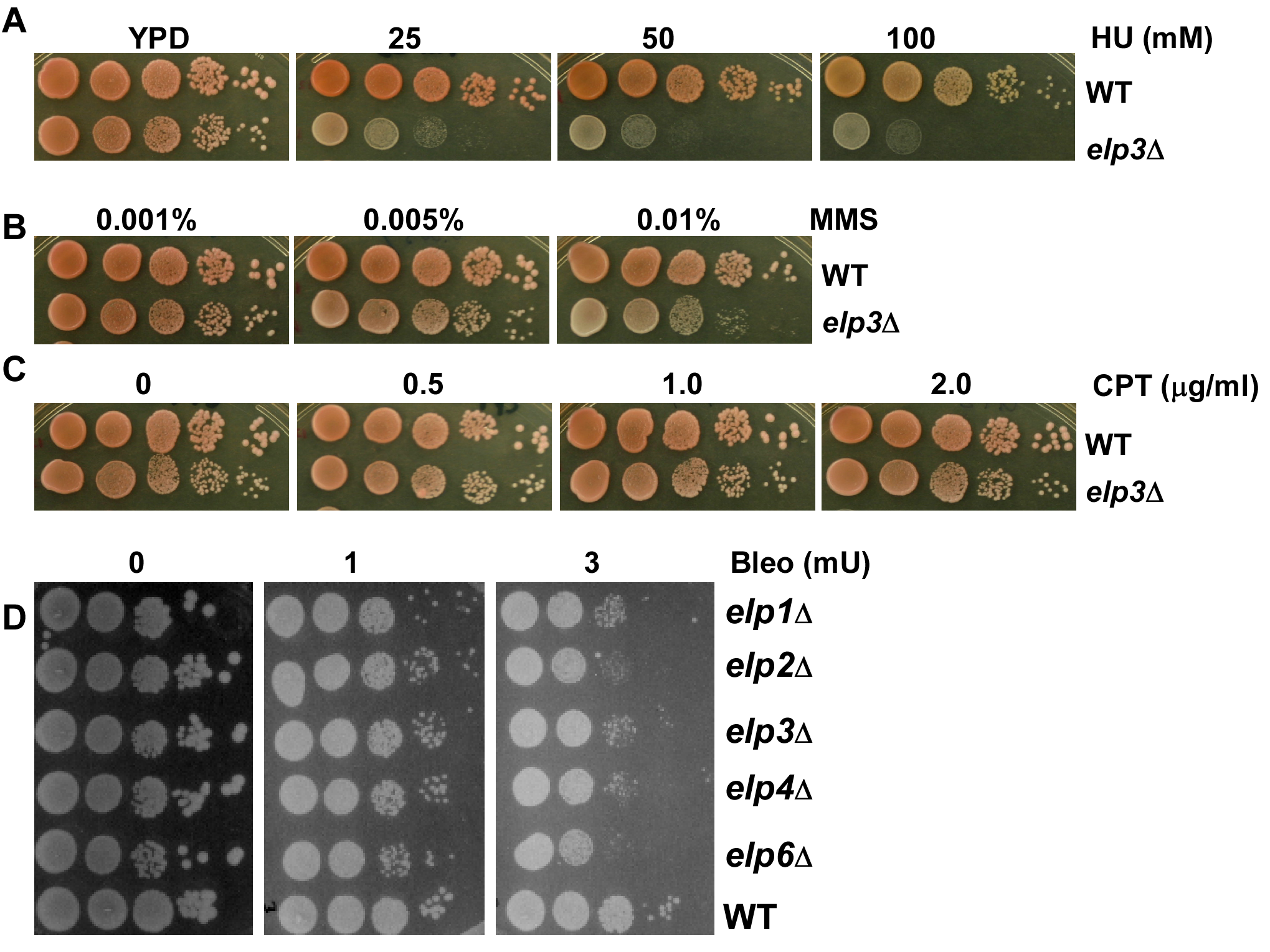

Supplement: Figure S1 — The ELP3 complex is important for resistance to DNA damaging agents. Ten fold series dilution of wild type or elp3Δ mutant cells were spotted onto medium containing different concentration of hydroxyurea (HU, A), methyl methane sulfate (MMS, B) or camptothecin (CPT, C). (D) Other subunits of the holo-elongator complex are required for the resistance to DNA damaging agents. Ten-fold serial dilutions of wild type or mutants lacking each of the five subunits of the holo-elongator complex were plated on medium containing different concentrations of bleomycin. The plates were incubated at 30°C for three days before pictures were taken. (1.74 MB TIF) [file pgen.1000684.s001.tif]

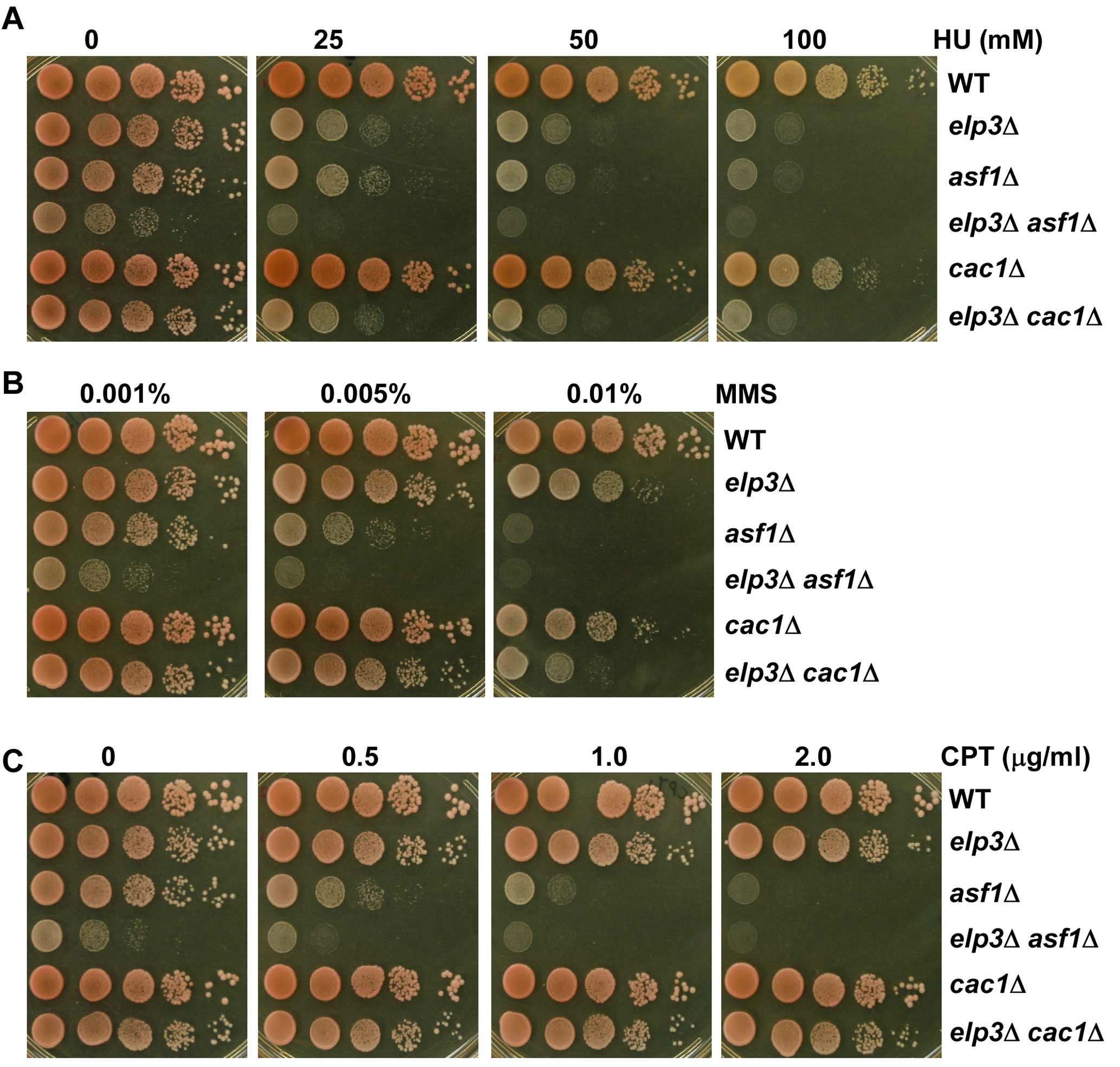

Supplement: Figure S2 — The elp3Δ mutation exhibits synthetic genetic interactions with asf1Δ but not cac1Δ mutant in response to DNA damaging agents. Ten-fold serial dilutions of yeast cells with relevant genotype identified at the right were spotted onto medium containing different HU (A), MMS (B) and CPT (C). Pictures were taken after three days of incubation at 30°C. (4.07 MB TIF) [file pgen.1000684.s002.tif]

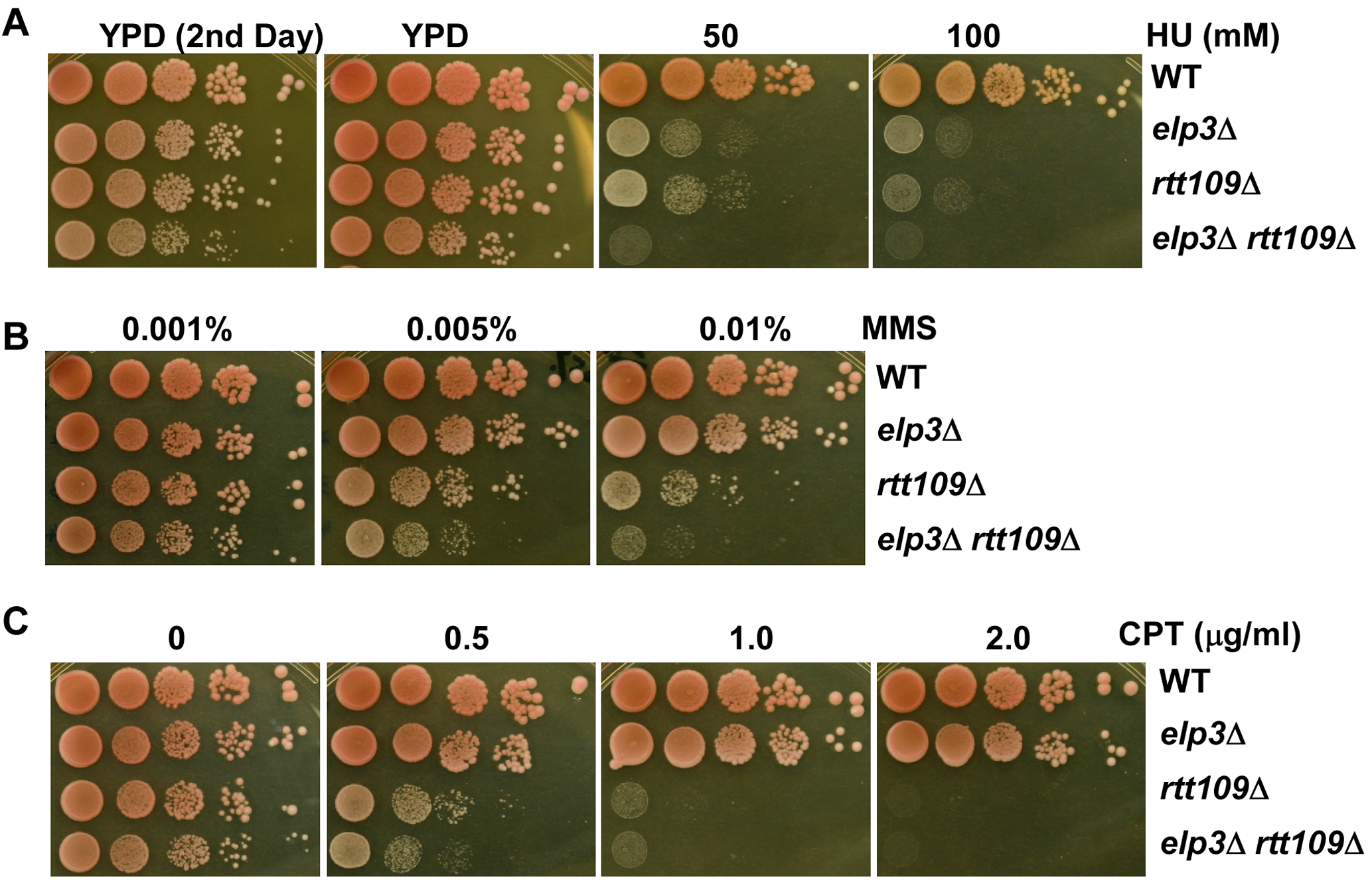

Supplement: Figure S3 — The elp3Δ rtt109Δ double mutant cells exhibit similar phenotypes to those of elp3Δ asf1Δ double mutant cells. Ten fold serial dilution of yeast cells with relevant genotype shown at the right were assayed for their sensitivity towards HU (A), MMS (B) or CPT (C). Images for cells growing on YPD plates were taken after incubation at 30°C for four days except that those indicated. (2.52 MB TIF) [file pgen.1000684.s003.tif]

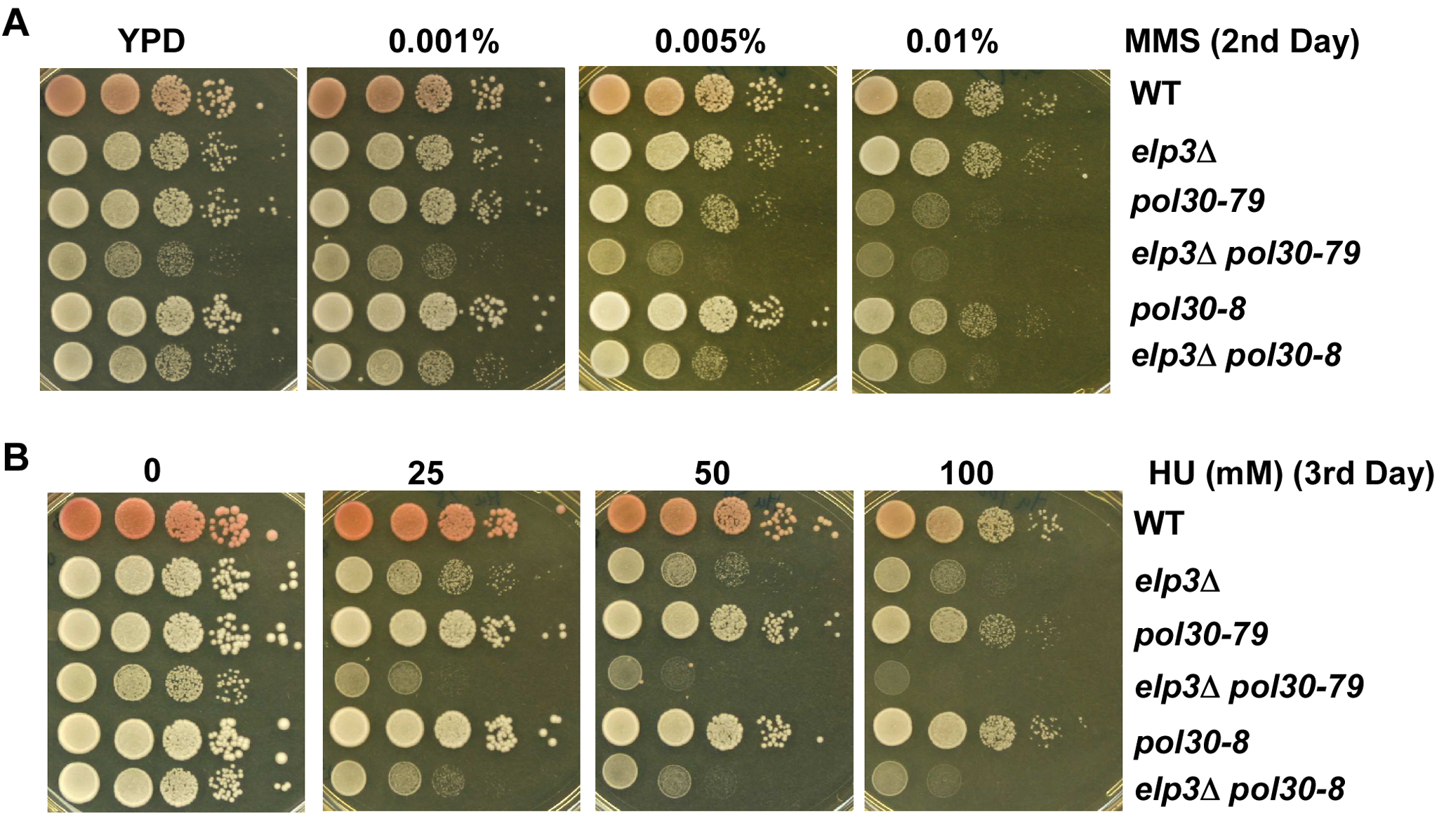

Supplement: Figure S4 — The elp3Δ mutant cells are more sensitive to DNA damage agents when combined with a PCNA mutant allele. Ten-fold serial dilution of yeast cells with relevant genotype shown at the right were assayed for their sensitivity towards MMS (A) or HU (B). Images for cells growing on YPD plates were taken after incubation at 30°C for three days. (2.35 MB TIF) [file pgen.1000684.s004.tif]
