# Supplementary material for: The Elongator Complex Interacts with PCNA and Modulates Transcriptional Silencing and Sensitivity to DNA Damage Agents
Source: PLoS Genet. 2009 Oct 16;5(10):e1000684. doi: 10.1371/journal.pgen.1000684 (PMC2757915; doi:10.1371/journal.pgen.1000684)
Supplement: Table S1 — The elp3Δ mutant does not exhibit synergistic loss of silencing at HMR locus in the absence of Asf1 using hmr::GFP silencing assay. The GFP transgene was integrated at the HMR locus in each strain with relevant genotype listed in the first column. Expression of GFP in wild type or various mutants was determined by FACS. Percentage of GFP expressing cells of each strain was shown. Because autofluorescence influenced the results significantly when the percentage of GFP positive cells was less than 1%, and we regarded the effect of any strains with less than 1% GFP cells as negligible. (0.04 MB DOC) [file pgen.1000684.s005.doc]

Table S1. The *elp3∆* mutant does not exhibit synergistic loss of silencing at *HMR* locus in the absence of Asf1 using *hmr::GFP* silencing assay.

| Genotype | GFP (%) |
| --- | --- |
| WT | < 1 |
| *sir3Δ* | 99.22 |
| *elp3Δ* | < 1 |
| *asf1Δ* | < 1 |
| *rtt109Δ* | < 1 |
| *cac1Δ* | < 1 |
| *rtt106Δ* | < 1 |
| *elp3Δ asf1Δ* | < 1 |
| *elp3Δ cac1Δ* | < 1 |
| *elp3Δ rtt106Δ* | < 1 |
| *elp3Δ rtt109Δ* | < 1 |
| *cac1Δ rtt106Δ* | 88.0 |
| *pol30-8* | < 1 |
| *pol30-79* | < 1 |
| *elp3△ pol30-8* | < 1 |
| *elp3△ pol30-79* | < 1 |

The GFP transgene was integrated at the *HMR* locus in each strain with relevant genotype listed in the first column. Expression of *GFP* in wild type or various mutants was determined by *FACS.* Percentage of GFP expressing cells of each strain was shown. Because autofluorescence influenced the results significantly when the percentage of GFP positive cells was less than 1%, and we regarded the effect of any strains with less than 1% GFP cells as negligible
