# Supplementary material for: The Elongator Complex Interacts with PCNA and Modulates Transcriptional Silencing and Sensitivity to DNA Damage Agents
Source: PLoS Genet. 2009 Oct 16;5(10):e1000684. doi: 10.1371/journal.pgen.1000684 (PMC2757915; doi:10.1371/journal.pgen.1000684)
Supplement: Table S2 — Yeast strains used in this study. (0.09 MB DOC) [file pgen.1000684.s006.doc]

Table S2: Yeast strains used in this study

| Name | Genetic background | Reference |
| --- | --- | --- |
| W3031A | *MATa leu2-3, 112 ura3-1 his3-11,15, trp1-1, ade2-1, can1-100* | 1 |
| YB1010 | *MATa leu2-3, 112 ura3-1 his3-11,15, trp1-1, ade2-1, can1-100, elp1Δ::kanMX6, URA3-VIIL* | This study |
| YB1012 | *MATa leu2-3, 112 ura3-1 his3-11,15, trp1-1, ade2-1, can1-100, elp2Δ::kanMX6, URA3-VIIL* | This study |
| YB1014 | *MATa leu2-3, 112 ura3-1 his3-11,15, trp1-1, ade2-1, can1-100, elp4Δ::kanMX6, URA3-VIIL* | This study |
| YB943 | *MATa leu2-3, 112 ura3-1 his3-11,15, trp1-1, ade2-1, can1-100, elp3Δ::kanMX6, URA3-VIIL* | This study |
| YB1016 | *MATa leu2-3, 112 ura3-1 his3-11,15, trp1-1, ade2-1, can1-100, elp6Δ::kanMX6, URA3-VIIL* | This study |
| YB1000 | *MATa leu2-3, 112 ura3-1 his3-11,15, trp1-1, ade2-1, can1-100, elp1-TAP::TRP* | This study |
| YB1001 | *MATa leu2-3, 112 ura3-1 his3-11,15, trp1-1, ade2-1, can1-100, elp3-TAP::TRP1* | This study |
| YB1002 | *MATa leu2-3, 112 ura3-1 his3-11,15, trp1-1, ade2-1, can1-100, elp2-TAP::TRP1* | This study |
| YB1003 | *MATa leu2-3, 112 ura3-1 his3-11,15, trp1-1, ade2-1, can1-100, elp4-TAP::TRP1* | This study |
| YB1007 | *MATa leu2-3, 112 ura3-1 his3-11,15, trp1-1, ade2-1, can1-100, elp5-TAP::TRP1* | This study |
| YB1065 | *MATa leu2-3, 112 ura3-1 his3-11,15, trp1-1, ade2-1, can1-100, hht1-hhf1::leu2,hht2-hhf2::kanMX6, HHT2-HHF2/pRS414,* | This study |
| YB1033 | *MATa leu2-3, 112 ura3-1 his3-11,15, trp1-1, ade2-1, can1-100, hht1-hhf1::leu2,hht2-hhf2::kanMX6, HHT2-HHF2/pRS414, elp3Δ::ADE2* | This study |
| YB1029 | *MATa leu2-3, 112 ura3-1 his3-11,15, trp1-1, ade2-1, can1-100, hht1-hhf1::leu2,hht2-hhf2::kanMX6, HHT2(K14G)-HHF2/pRS414* | This study |
| YB1034 | *MATa leu2-3, 112 ura3-1 his3-11,15, trp1-1, ade2-1, can1-100, hht1-hhf1::leu2,hht2-hhf2::kanMX6, HHT2(K14G)-HHF2/pRS414, elp3Δ::ADE2* | This study |
| YB1031 | *MATa leu2-3, 112 ura3-1 his3-11,15, trp1-1, ade2-1, can1-100, hht1-hhf1::leu2,hht2-hhf2::kanMX6, HHT2(K14Q)-HHF2/pRS414* | This study |
| YB1035 | *MATa leu2-3, 112 ura3-1 his3-11,15, trp1-1, ade2-1, can1-100, hht1-hhf1::leu2,hht2-hhf2::kanMX6, HHT2(K14Q)-HHF2/pRS414, elp3Δ::ADE2* | This study |
| YB1030 | *MATa leu2-3, 112 ura3-1 his3-11,15, trp1-1, ade2-1, can1-100, hht1-hhf1::leu2,hht2-hhf2::kanMX6, HHT2(K9,14,18,23,27R)-HHF2/pRS414* | This study |

| Name | Genetic background | Reference |
| --- | --- | --- |
| ZGY346 | *MATa leu2-3, 112 ura3-1 his3-11,15, trp1-1, ade2-1, can1-100,elp3Δ::kanMX6, elp5-TAP::TRP1* | This study |
| YB1036 | *MATa leu2-3, 112 ura3-1 his3-11,15, trp1-1, ade2-1, can1-100, hht1-hhf1::leu2,hht2-hhf2::kanMX6, HHT2(K9,14,18,23,27R)-HHF2/pRS414, elp3Δ::ADE2* | This study |
| YB1032 | *MATa leu2-3, 112 ura3-1 his3-11,15, trp1-1, ade2-1, can1-100, hht1-hhf1::leu2,hht2-hhf2::kanMX6, HHT2(K14R)-HHF2/pRS414* | This study |
| YB1037 | *MATa leu2-3, 112 ura3-1 his3-11,15, trp1-1, ade2-1, can1-100, hht1-hhf1::leu2, hht2-hhf2::kanMX6,*  *HHT2(K14R)-HHF2/pRS414, elp3Δ::ADE2* | This study |
| ZGY300 | *MATa leu2-3, 112 ura3-1 his3-11,15, trp1-1, ade2-1, can1-100, elp3Δ::kanMX6, cac1Δ::LEU, URA3-VIIL* | This study |
| ZGY689 | *MATa leu2-3, 112 ura3-1 his3-11,15, trp1-1, ade2-1, can1-100, elp3Δ::kanMX6, rtt106Δ::natR, URA3-VIIL* | This study |
| ZGY691 | *MATa leu2-3, 112 ura3-1 his3-11,15, trp1-1, ade2-1, can1-100, elp3Δ::kanMX6, asf1Δ::natR, URA3-VIIL* | This study |
| ZGY304 | *MATa leu2-3, 112 ura3-1 his3-11,15, trp1-1, ade2-1, can1-100, elp3Δ::kanMX6, elp3-Flag::TRP1, hmr::ADE2, URA3-VIIL* | This study |
| ZGY306 | *MATa leu2-3, 112 ura3-1 his3-11,15, trp1-1, ade2-1, can1-100, elp3Δ::kanMX6, elp3-1-Flag::TRP1, hmr::ADE2, URA3-VIIL (C108C110AA)* | This study |
| ZGY308 | *MATa leu2-3, 112 ura3-1 his3-11,15, trp1-1, ade2-1, can1-100, elp3Δ::kanMX6, elp3-2-Flag::TRP1, hmr::ADE2, URA3-VIIL (C118C121AA)* | This study |
| ZGY310 | *MATa leu2-3, 112 ura3-1 his3-11,15, trp1-1, ade2-1, can1-100, elp3Δ::kanMX6, elp3-3-Flag::TRP1, hmr::ADE2, URA3-VIIL (G180G181RR)* | This study |
| ZGY312 | *MATa leu2-3, 112 ura3-1 his3-11,15, trp1-1, ade2-1, can1-100, elp3Δ::kanMX6, elp3-4-Flag::TRP1, hmr::ADE2, URA3-VIIL (G168R)* | This study |
| ZGY314 | *MATa leu2-3, 112 ura3-1 his3-11,15, trp1-1, ade2-1, can1-100, elp3Δ::kanMX6, elp3-5-Flag::TRP1, hmr::ADE2, URA3-VIIL (Y540Y541AA)* | This study |
| ZGY423 | *MATa leu2-3, 112 ura3-1 his3-11,15, trp1-1, ade2-1, can1-100, elp3Δ::kanMX6, elp1-TAP::TRP1* | This study |
| ZGY424 | *MATa leu2-3, 112 ura3-1 his3-11,15, trp1-1, ade2-1, can1-100, elp3Δ::kanMX6, elp2-TAP::TRP1* | This study |
| ZGY810 | *MATa leu2-3, 112 ura3-1 his3-11,15, trp1-1, ade2-1, can1-100, hap2Δ::kanMX6, URA3-VIIL* | This study |
| ZGY484 | *MATα leu2-3, 112 ura3-1 his3-11,15, trp1-1, ade2-1, can1-100, rtt106Δ:natR* | This study |

| Name | Genetic background | | Reference |
| --- | --- | --- | --- |
| ZGY813 | *MATa leu2-3, 112 ura3-1 his3-11,15, trp1-1, ade2-1, can1-100, asf1Δ:natR, hap2Δ::kanMX6, URA3-VIIL* | | This study |
| ZGY821 | *MATa leu2-3, 112 ura3-1 his3-11,15, trp1-1, ade2-1, can1-100, asf1Δ:natR, hap3Δ::kanMX6,URA3-VIIL* | | This study |
| ZGY584 | *MATa leu2-3, 112 ura3-1 his3-11,15, trp1-1, ade2-1, can1-100, cac1Δ:LEU* | | This study |
| ZGY1493 | *MATa leu2-3, 112 ura3-1 his3-11,15, trp1-1, ade2-1, can1-100, rtt109Δ:natR, hmr::GFP* | | This study |
| ZGY1494 | *MATa leu2-3, 112 ura3-1 his3-11,15, trp1-1, ade2-1, can1-100, rtt109Δ:natR, elp3Δ::kanMX6, hmr::GFP* | | This study |
| ZGY1489 | *MATa leu2-3, 112 ura3-1 his3-11,15, trp1-1, ade2-1, can1-100, hht1-hhf1::leu2, hht2-hhf2::kanMX6,*  *HHT2-HHF2/yCP50, elp3Δ::ADE2* | | This study |
| ZGY1490 | *MATa leu2-3, 112 ura3-1 his3-11,15, trp1-1, ade2-1, can1-100, hht1-hhf1::leu2, hht2-hhf2::kanMX6,*  *HHT2-HHF2(K5,8,12R)/pRS313, elp3Δ::ADE2* | | This study |
| ZGY1491 | *MATa leu2-3, 112 ura3-1 his3-11,15, trp1-1, ade2-1, can1-100, hht1-hhf1::leu2, hht2-hhf2::kanMX6,*  *HHT2(H3K56R)-HHF2/pRS313, elp3Δ::ADE2* | | This study |
| ZGY1492 | *MATa leu2-3, 112 ura3-1 his3-11,15, trp1-1, ade2-1, can1-100, hht1-hhf1::leu2, hht2-hhf2::kanMX6,*  *HHT2-HHF2(K5,12R)pRS414, elp3Δ::ADE2* | | This study |
| ZGY1698 | *MATa leu2-3, 112 ura3-1 his3-11,15, trp1-1, ade2-1, can1-100, hht1-hhf1::leu2, hht2-hhf2::kanMX6,*  *HHT2-HHF2(K8R/)pRS313, URA3-VIIL* | | This study |
| ZGY1699 | *MATa leu2-3, 112 ura3-1 his3-11,15, trp1-1, ade2-1, can1-100, hht1-hhf1::leu2, hht2-hhf2::kanMX6,URA3-VIIL*  *HHT2-HHF2(K8R)/pRS313, elp3Δ::ADE2* | | This study |
| ZGY1641 | *MATa leu2-3, 112 ura3-1 his3-11,15, trp1-1, ade2-1, can1-100, hht1-hhf1::leu2, hht2-hhf2::kanMX6,*  *elp3Δ::ADE2, pol30-79::natR, hmr::GFP* | | This study |
| ZGY1645 | *MATa leu2-3, 112 ura3-1 his3-11,15, trp1-1, ade2-1, can1-100, hht1-hhf1::leu2, hht2-hhf2::kanMX6,*  *elp3Δ::ADE2, pol30-8::natR, hmr::GFP* | | This study |
| ZGY444 | *MATa leu2-3, 112 ura3-1 his3-11,15, trp1-1, ade2-1, can1-100, hht1-hhf1::leu2, hht2-hhf2::kanMX6,*  *pol30-8::natR, hmr::GFP* | | This study |
| ZGY467 | *MATa leu2-3, 112 ura3-1 his3-11,15, trp1-1, ade2-1, can1-100, hht1-hhf1::leu2, hht2-hhf2::kanMX6,*  *pol30-79::natR, hmr::GFP* | | This study |
| ZGY1069 | *MATa leu2-3, 112 ura3-1 his3-11,15, trp1-1, ade2-1, can1-100, hht1-hhf1::leu2, hht2-hhf2::kanMX6,*  *HHT2-HHF2(K5,8,12R)/pRS313* | | 2 |
| Name | Genetic background | Reference | |
| ZGY853 | *MATa leu2-3, 112 ura3-1 his3-11,15, trp1-1, ade2-1, can1-100, hht1-hhf1::leu2, hht2-hhf2::kanMX6,*  *HHT2(H3K56R)-HHF2/pRS313, elp3Δ::ADE2* | 2 | |
| ZGY1087 | *MATa leu2-3, 112 ura3-1 his3-11,15, trp1-1, ade2-1, can1-100, hht1-hhf1::leu2, hht2-hhf2::kanMX6,*  *HHT2-HHF2(K5,12R)pRS414, elp3Δ::ADE2* | 2 | |
